# Docetaxel-loaded solid lipid nanoparticles prevent tumor growth and lung metastasis of 4T1 murine mammary carcinoma cells

**DOI:** 10.1186/s12951-020-00604-7

**Published:** 2020-03-12

**Authors:** Márcia Cristina Oliveira da Rocha, Patrícia Bento da Silva, Marina Arantes Radicchi, Bárbara Yasmin Garcia Andrade, Jaqueline Vaz de Oliveira, Tom Venus, Carolin Merker, Irina Estrela-Lopis, João Paulo Figueiró Longo, Sônia Nair Báo

**Affiliations:** 1 Electron Microscopy Laboratory, Institute of Biological Sciences, University of Brasilia, Brasília, Brazil; 2 Nanobiotechnology Laboratory, Institute of Biological Sciences, University of Brasilia, Brasília, Brazil; 3 Institute of Medical Physics & Biophysics, Leipzig University, Leipzig, Germany

**Keywords:** Cellular uptake, 4T1, NIH-3T3, IL-6, BCL-2, Ki-67 and antitumor effect

## Abstract

**Background:**

Metastasis causes the most breast cancer-related deaths in women. Here, we investigated the antitumor effect of solid lipid nanoparticles (SLN-DTX) when used in the treatment of metastatic breast tumors using 4T1-bearing BALB/c mice.

**Results:**

Solid lipid nanoparticles (SLNs) were produced using the high-energy method. Compritol 888 ATO was selected as the lipid matrix, and Pluronic F127 and Span 80 as the surfactants to stabilize nanoparticle dispersion. The particles had high stability for at least 120 days. The SLNs’ dispersion size was 128 nm, their polydispersity index (PDI) was 0.2, and they showed a negative zeta potential. SLNs had high docetaxel (DTX) entrapment efficiency (86%), 2% of drug loading and showed a controlled drug-release profile. The half-maximal inhibitory concentration (IC_50_) of SLN-DTX against 4T1 cells was more than 100 times lower than that of free DTX after 24 h treatment. In the cellular uptake test, SLN-DTX was taken into the cells significantly more than free DTX. The accumulation in the G2-M phase was significantly higher in cells treated with SLN-DTX (73.7%) than in cells treated with free DTX (23.0%), which induced subsequent apoptosis. TEM analysis revealed that SLN-DTX internalization is mediated by endocytosis, and fluorescence microscopy showed DTX induced microtubule damage. In vivo studies showed that SLN-DTX compared to free docetaxel exhibited higher antitumor efficacy by reducing tumor volume (p < 0.0001) and also prevented spontaneous lung metastasis in 4T1 tumor-bearing mice. Histological studies of lungs confirmed that treatment with SLN-DTX was able to prevent tumor. IL-6 serum levels, ki-67 and BCL-2 expression were analyzed and showed a remarkably strong reduction when used in a combined treatment.

**Conclusions:**

These results indicate that DTX-loaded SLNs may be a promising carrier to treat breast cancer and in metastasis prevention.
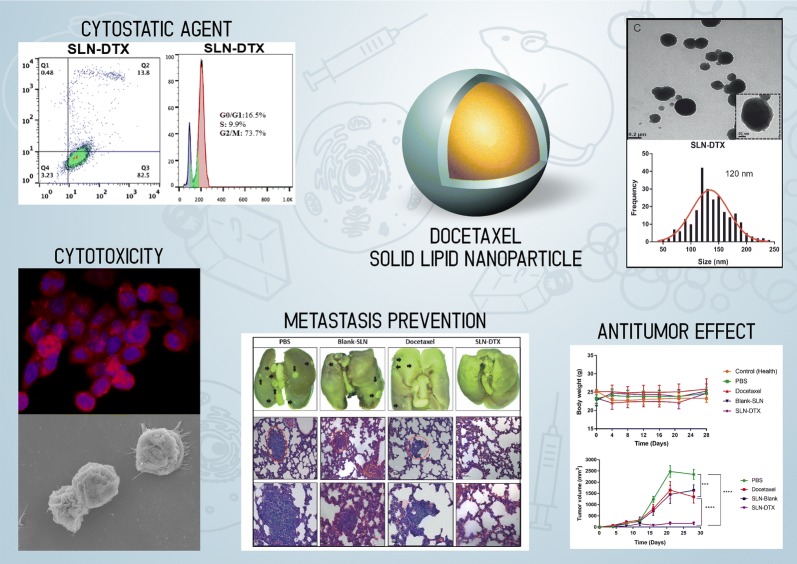

## Background

Breast cancer is the most prevalent malignant tumor in women and a major cause of mortality among women worldwide. Metastasis causes the majority of breast cancer-related deaths. Chemotherapy is used to prolong patient survival and prevent recurrence and metastasis [1]. Taxanes (docetaxel or paclitaxel) are examples of chemotherapies that are used in these contexts [2].

Docetaxel (DTX) is a lipophilic anticancer agent and a semi-synthetic taxane derived from the European tree *Taxus baccata* [3]. Docetaxel has been approved by the Food and Drug Administration (FDA) and is widely used for different types of cancer, such as breast cancer, ovarian cancer, prostate cancer, non-small-cell lung cancer, gastric adenocarcinoma, and others [4]. DTX acts by binding reversibly to microtubules, promoting transitory structure stabilization, leading to cell cycle arrest. Therefore, docetaxel is a cytostatic drug for the control of tumor tissue growth [5].

In terms of clinical importance, taxane has an important role in metastatic breast cancer treatment. DTX showed some improved survival outcomes regarding metastatic disease when compared with other chemotherapeutic agents [6]. However, the clinical administration of intravenous DTX has been limited due to its poor aqueous solubility (4.93 µg/mL in purified water), high lipophilicity (logP = 4.1), low bioavailability and high toxicity. To increase the solubility of DTX, the pharmaceutical industry developed some formulations containing surfactants, such as Tween-80, and/or alcohol, to combat these pharmacotechnical problems. Nevertheless, as highly reactive components, these formulations cause some adverse reactions in patients, including hypersensitivity, neurotoxicity, musculoskeletal toxicity and fluid retention [7]. In order to reduce these side effects, researchers are developing different types of drug delivery systems (DDS), such as nanoparticles (NPs), to overcome these drawbacks related to DTX. Drug delivery systems, such as solid lipid nanoparticles [8], liposomes [9], nanoemulsions [10], and polymeric micelles [11] could improve DTX’s therapeutic effect, increase stability, and boost drug biocompatibility.

Among the different types of lipid nanostructures, solid lipid nanoparticles (SLNs) are an attractive DDS due to their high structural stability and biocompatibility in comparison to nanoemulsions and are considered a less toxic alternative to polymer-based nanoparticles [12]. SLNs are made from physiologically tolerable lipid components, which remain in the solid state at room and body temperature [13]. Some advantages of SLNs include low toxicity, controlled drug release, physical stability, large-scale production, protection of incorporated drug, high drug loading, low cost, avoidance of organic solvents in preparation, biodegradability, biocompatibility and capability of incorporating hydrophilic and hydrophobic compounds [14, 15]. Thus, the development of docetaxel-loaded SLNs (SLN-DTX) provides a good alternative carrier to prolong DTX plasma circulation half-life, prevent drug toxicity and increase DTX delivery to tumor tissues [16].

Therefore, in accordance with the literature review presented, the aim of this study was to evaluate the efficiency of SLN-DTX in vitro and in vivo in a tumor xenografts model induced in female BALB/C mice.

## Results and discussion

Docetaxel is one of the most important chemotherapeutical drugs available nowadays. Its best-known pharmaceutical form is Taxotere©, which is a product containing docetaxel and two excipients ingredients, ethanol and tween 80. This is a successful product with billionaire revenues, however some improvements on the product formulation are required by the medical community. It is a consensus that ethanol administration should be avoid to reduce the potential toxicity of the formulation. Thus, several research centers are investing efforts to produce a commercially viable Docetaxel formulation that could overcome the actual formulations drawbacks.

Our research group developed different types of nanocarriers that could be useful for this propose. For the present article, we design a Solid Lipid Nanoparticle that could efficiently entrap Docetaxel without any organic solvent, and that the preparation process was carried out entirely in aqueous systems, aiming the industrial scale-up process. As highlight of our results, we could say that the SLN developed had an encapsulation rate higher than 85%, result that is difficult to be reproduced in other carrier systems, such as liposomes. In addition, as presented in our results, this Solid Lipid Nanoparticle had excellent results in terms of tumor control and prevention of distant lung metastasis. This second is really important to highlight because the experiments were conduced in a 4T1 tumor bearing mice, which has a strong metastatic profile, and we consider a differential result in comparison to others docetaxel nanocarriers.

### Preparation, characterization and in vitro release of SLNs

**Fig. 1:**
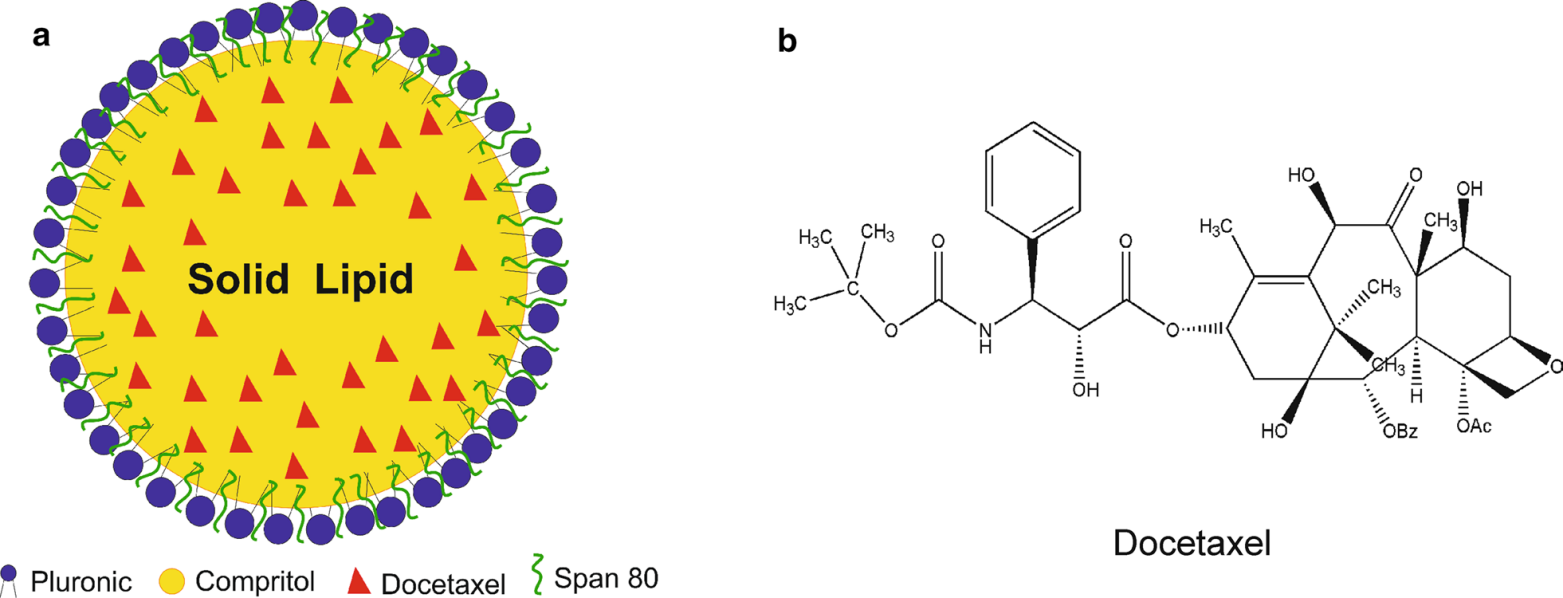
**a** Schematic illustration of SLN-DTX. **b** Chemical structure of docetaxel (DTX)

Solid lipid nanoparticles were prepared by high-energy method. Compritol was selected as the solid lipid, and the selected surfactants were Span 80 and Pluronic F127. This lipid phase was chosen based on some reports in the literature [17–19], and the final formulation was established as the best formulation that remained stable after the incorporation of docetaxel. The use of two surfactants combined (in this study we used Span 80 and Pluronic F127) instead of only one is more effective, reducing the interfacial tension in nanoparticles to prevent their aggregation, producing smaller and more stable nanoparticles [20]. A study comparing Pluronic F68 and Pluronic F127 showed that nanoparticles made with Pluronic F127 produce a stronger hydrophobic interaction with DTX than when made with Pluronic F68. This is due to the lower HLB (hydrophilic–lipophilic balance) value of Pluronic F127. It results in a better suspension preparation and could prevent nanoparticle aggregation [21]. Thus, it is important to choose suitable surfactants for SLN preparations. Figure 1a shows the schematic model illustration of docetaxel-loaded solid nanoparticles and Fig. 1b shows the docetaxel structure. The model was based on the lipophilicity of docetaxel, which in theory would be dispersed in the SLNs’ lipid core [14, 22].

The particles’ charge interaction with medium is measured as the zeta potential parameter. It is considered as one reference for colloidal stability. As a repellent surface force, this parameter contributes to keeping the dispersed nanoparticles separate, thereby assisting in the formulation stability [23]. The zeta potential value of blank-SLN and SLN-DTX were − 11 and − 15 mV, respectively. There were no significant differences in zeta potential value between blank and SLN-DTX, suggesting that DTX incorporation did not alter colloidal stability. The negative surface charge induces electrostatic repulsion. It can ensure the physical stability of nanoparticles during storage and can prevent the formation of aggregates [24]. In addition, negative charged particles tend to have an increased plasma circulation time [25, 26]. The mean hydrodynamic diameter of the SLN-DTX was 128 ± 2.2 nm with a polydispersity index of 0.153 ± 0.02. The assessment of the colloidal stability in physiological fluids is important. Thus, we performed the SLN-DTX interaction with biological media (Additional file 1: Figure S1). The DH, PDI and zeta potential remain stable at sample up to 7 days after formulation, with the exception of zeta potential in serum (p > *0.1), as result of protein aggregation and corona formation. When nanoparticles are added to serum they enter in contact with albumin and other proteins, forming the protein corona, and protein adsorption strongly occurs. Some authors have demonstrated that the formation of protein layer destabilizes the nanoparticles, which affects the surface charge distribution [27].

**Fig. 2:**
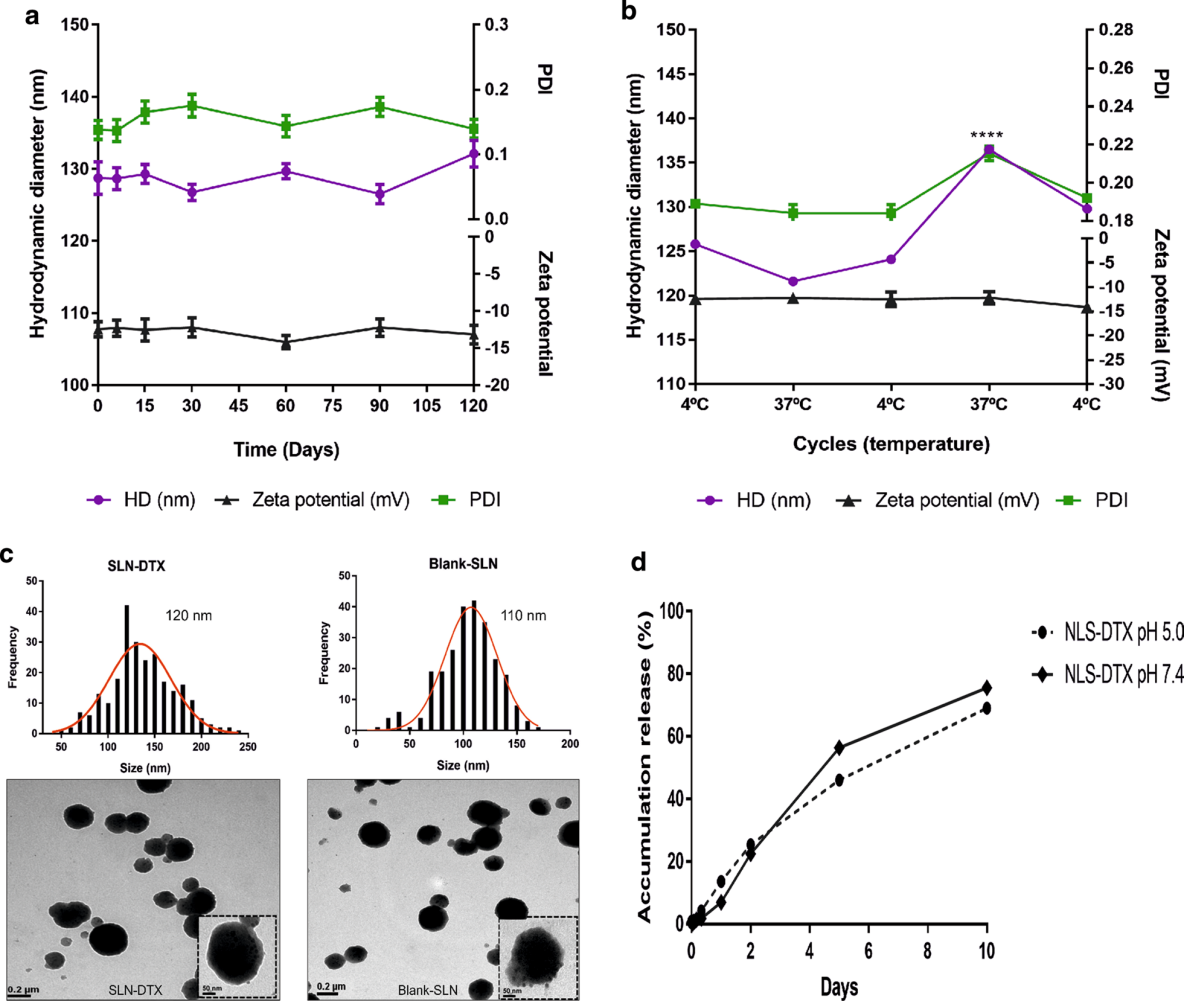
**a** Colloidal stability of SLN-DTX over 120 days. Hydrodynamic diameter (HD), zeta potential and polydispersity index (PDI) measured by dynamic light scattering. **b** Storage stability study of SLN-DTX. HD, zeta potential and PDI index were analyzed after samples were incubated for 24 h at 4 °C or 37 °C (n = 3). **c** Nanoparticle size distribution of SLN-DTX had an average size of 120 nm and blank-SLN around 110 nm; measurements were performed using ImageJ software. Morphology of SLN-DTX and blank-SLN was assessed by transmission electron microscopy (TEM) at 100 K and in dashed line 250 K magnification. **d** Drug release profiles from SLN-DTX at pH 7.4 and pH 5.0 PBS at 10 days. Data are expressed as mean ± standard error of the mean (****p < 0.0001)

Studies demonstrate that PDI values lower than 0.2 indicate a homogeneous distribution and uniformity of nanoparticles [28, 29]. Thus, zeta potential, size and PDI did not show significant changes, indicating that SLN-DTX are stable over the 120 days (Fig. 2a). Figure 2b shows the storage stability study of SLN-DTX. Mean particle size was 126 ± 5.0 nm with PDI of 0.19 ± 0.01, and zeta potential was − 15 ± 0.5 mV. These results demonstrate the suitable stability of SLN-DTX, but with a significant change only during the fourth measurement of particle size (p**** < 0.0001). After being subjected to this stress test, samples remained stable after abrupt temperature change.

Medium-sized nanoparticles of less than 500 nm with a spherical shape have enhanced internalization efficiency in the cell. Spherical particles have the right shape that allows them to attach onto the cells and offer a high volume of the incorporated drug [30]. According to TEM micrographs, blank-SLN and SLN-DTX are spherical and uniform, which demonstrated that the entrapment of DTX would not significantly influence the shape of the SLNs. The mean particle size of blank-SLNs is 110 nm, and of SLN-DTX is 120 nm (Fig. 2c). These results are in agreement with DLS measurements.

Entrapment efficiency and the drug loading of SLN-DTX were 86 ± 2.4% and 2% ± 0.12%, respectively. The EE% and DL% depends on the composition of the lipid matrix and its crystalline state. It has been reported that in lipid-based drug delivery systems, the binding energy of the drugs with the lipids plays a key role in successful encapsulation of drugs [31]. The formulation (SLN-DTX) presented similar entrapment efficiency and drug loading comparing to works previously reported in the literature [32, 33].

The in vitro drug release profiles of SLN-DTX were evaluated at the pH values of 7.4 and 5.0 to mimic the physiological pH of blood and the acidic intracellular environment of the tumor cells, respectively (Fig. 2d). We identified an initial burst effect release, followed by controlled release, in SLN-DTX at both pHs. The SLN-DTX drug accumulation release percentages reached over 77% at pH 5.0 and 69% at pH 7.4 in 10 days, respectively. No differences were observed. The prolonged release suggests homogeneous entrapment of the DTX throughout the system. It was possible to observe this strong interaction in the results of DSC, Raman and FTIR. The benefit of the prolonged release system could be the reduction of the dose and drug administration by maintaining drug concentrations in a therapeutic window over a long period of time [34]. This profile affirms the applicability of SLNs as a promising drug carrier.

### Fourier transform infrared and raman spectroscopy

**Fig. 3:**
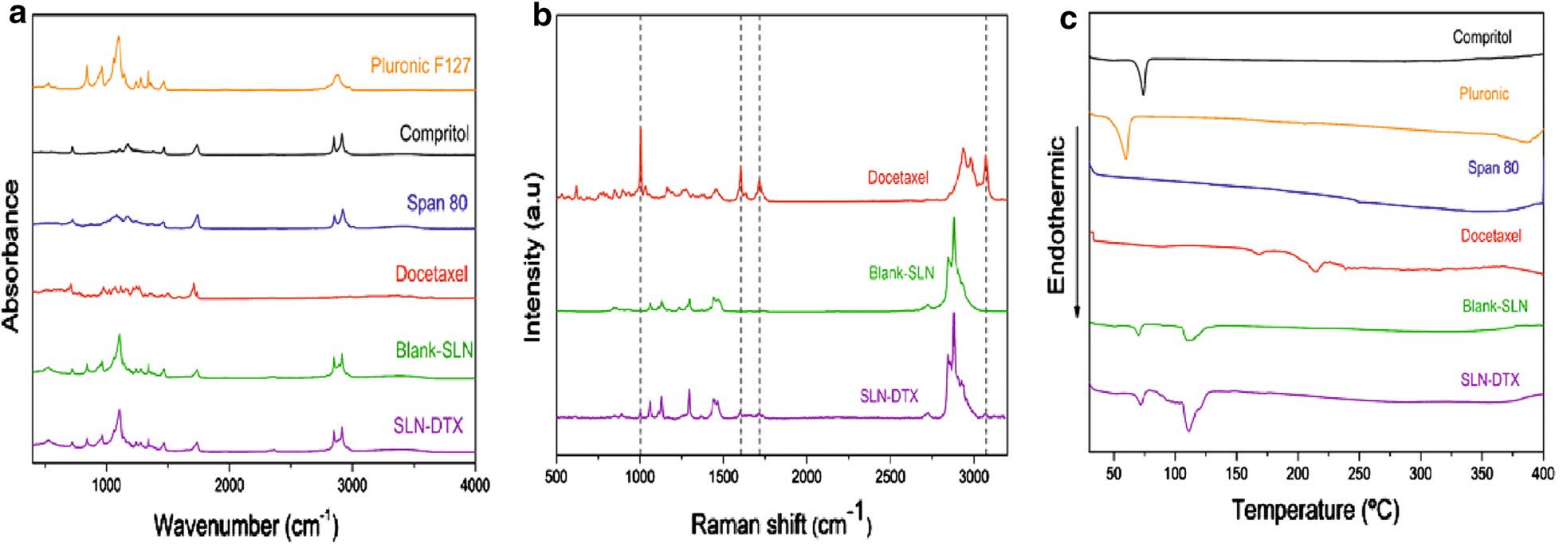
**a** Vibrational spectra in the infrared region of Pluronic F127, Compritol, Span 80, docetaxel, Blank-SLN and SLN-DTX. **b** Raman spectra of docetaxel. The highlights represent docetaxel bands in SLN-DTX spectra. **c** DSC thermograms

The spectroscopy technique was used in the infrared region to determine the interaction level of functional groups in the active material (docetaxel) and the carrier system. The FTIR assignments and frequencies are shown in Additional file 2: Table S1. Additional file 3: Figure S2 shows the FTIR spectrum of DTX. The FTIR spectra are given in Fig. 3-a for SLN-DTX, blank-SLN, free DTX and SLN components: Span 80, Compritol and Pluronic F127. The DTX FTIR spectrum showed bands at 3369 cm^−1^ (νO–H and νN–H), 1437 cm^−1^ (νC=C), 2982 cm^−1^ (ν_as_CH), 2937 cm^−1^ (ν_s_CH), 709 cm^−1^ (δCH), two bands assigned to the vibrational mode νC=0, one at 1738 cm^−1^ and another at 1709 cm^−1^, relative to carbonyl groups of ester and ketone, respectively [2]. Blank-SLN FTIR spectra were very similar to SLN-DTX analysis. The DTX characteristic bands were not observed in SLN-DTX FTIR spectra. The DTX is present in low concentrations in the NLS-DTX, and this makes it difficult to detect the bands assigned to the drug and to assess changes in the frequency of them, FTIR technique, therefore doesn’t confirm the presence of the drug in the carrier. Similar results were analyzed by Albano et al. [35] in a study that observed the absence of the DTX band in lipid-polymeric nanoparticle by FTIR technique. During preparation of the nanoparticles, there is a possibility of different physical–chemical interactions, such as the formation of hydrogen bonds between SLN components and drug. This interaction influences the bond stiffness and so alters the frequency of vibration [36, 37]. Solid lipids may be interacting with DTX groups. However, the FTIR spectrum of SLNs shows the characteristic peaks of similar functional groups, confirming the successful formulation of nanoparticles without any chemical interaction [38].

The Raman spectral signature of our DTX was in agreement with previous reports [39] (Additional file 4: Figure S3 and Additional file 5: Table S2). Figure 3b shows DTX spectral bands at 620 and 1006 cm^−1^, which are attributed to the aromatic vibrations of monosubstituted benzene, while vibrational bands at 1605, 1632, and 1715 cm^−1^ were respectively assigned to the ester, aromatic stretch (νC=C) and carbonyl groups present in the DTX drug. In the region of 1200–1430 cm^−1^, the secondary alcohol group is present. There are also broad bands with shoulders in the region of 1200–1500 cm^−1^, which are attributed to the deformation of CH_2_ and CH_3_ [40]. New bands appeared in the SLN-DTX spectra by comparison with the blank-SLN spectra (Fig. 3b). These bands correspond to the presence of docetaxel. Bands can be observed at 1006, 1605, 1715 and 3072 cm^−1^ of SLN-DTX spectra, and these are not observed in blank-SLN spectra. These bands are attributed to the vibrational modes νC=C (aromatic), νC=C with νC=O, νC=O of the carbonyl groups and ν (O–H). The presence of these DTX characteristic bands, even at a low intensity, permits us to infer that the drug is present in the lipid system (SLN-DTX).

Some peaks observed in DTX spectra decreased or are absent in SLN-DTX Raman spectra, which indicates that the crystalline structure of the drug was lost after incorporation into the carrier. The same was observed by Gao et al. [41], in a study which incorporated DTX into albumin-lipid nanoparticles, in which some DTX bands had reduced intensity or disappeared after incorporation into the nanoparticles.

### Differential scanning calorimetry—(DSC)

Differential scanning calorimetry is generally used to obtain information about physical and energy properties of a compound or formulation. Based on physical or chemical sample changes, the DSC technique measures the loss or gain of heat in samples as a function of temperature [42]. Thermograms of DTX, Compritol, Pluronic F127, Span 80, blank-SLN and SLN-DTX are shown in Fig. 3-c. The thermal data (melting point and ΔH) of the SLNs and DTX constituents are shown in Table 1.

**Table 1:** DSC of solid lipid nanoparticle constituents and docetaxel

Sample	T_peak_/°C	∆H (J/g)
Compritol	73.50	95.05
Pluronic	57.51	133.05
Span 80	129	34.60
Docetaxel	168.1	7.92

Thermal analysis of Compritol 888 ATO by DSC showed 73.5 °C as the endothermic peak, corresponding to the melting point of the compound in polymorph form β, the most stable form [17]. Rahman et al. [23], showed that the melting point of Compritol 888 ATO was also observed at 71.2 °C, indicating the crystalline nature of polymorph β. The presence of the endothermic melting peak of Compritol at 69 to 74 °C confirmed the solid state of the lipid core within Blank-SLN and SLN-DTX. The crystalline state of the lipid core is essential for the incorporation of the lipophilic drug and the sustained release properties of the lipid nanoparticles [34]. Pluronic F127 shows an endothermic peak that corresponds to melting at 57.51 °C, and a similar result was observed by Newa et al. [43] and Karolewicz et al. [18]. Indeed, the authors reported the Pluronic F127 endothermic event at 57.29 °C and 53.42 °C, respectively. Span 80 at room temperature (initial analysis temperature) is liquid and it is not possible to visualize the melting point in DSC. Initial Span 80 degradation is at 129 °C [19].

DSC curve of DTX shows an endothermic peak at 168.1 °C, corresponding to the drug’s melting point [44]. This endothermic peak of DTX were no longer present in DSC thermogram of SLN-DTX. This would suggest the prevalence of strong drug–lipid interactions, with the conversion of initially crystalline DTX into an amorphous state or disordered in lipid core [8, 45]. The absence of the characteristic DTX peaks in DSC was previously shown to be related to the loss of DTX crystallinity [8, 46]. Sanna et al. [47] studied polymeric particles containing DTX for prostate cancer treatment. In all nanoparticles analyzed, the authors observed that the DTX melting peak disappeared completely from the DSC curve, evidencing the absence of a crystalline drug in nanoparticle samples. Data from other previous studies also demonstrate that it is not possible to observe the peaks related to DTX fusion in the DSC curve of the nanoparticle formulation containing the drug [7, 45, 48–50]. Moreover, the slower release of DTX from SLN-DTX, as shown in the results of the in vitro study, may also be attributed to the strong DTX–lipid interaction.

### In vitro cytotoxicity studies—MTT assay

**Fig. 4:**
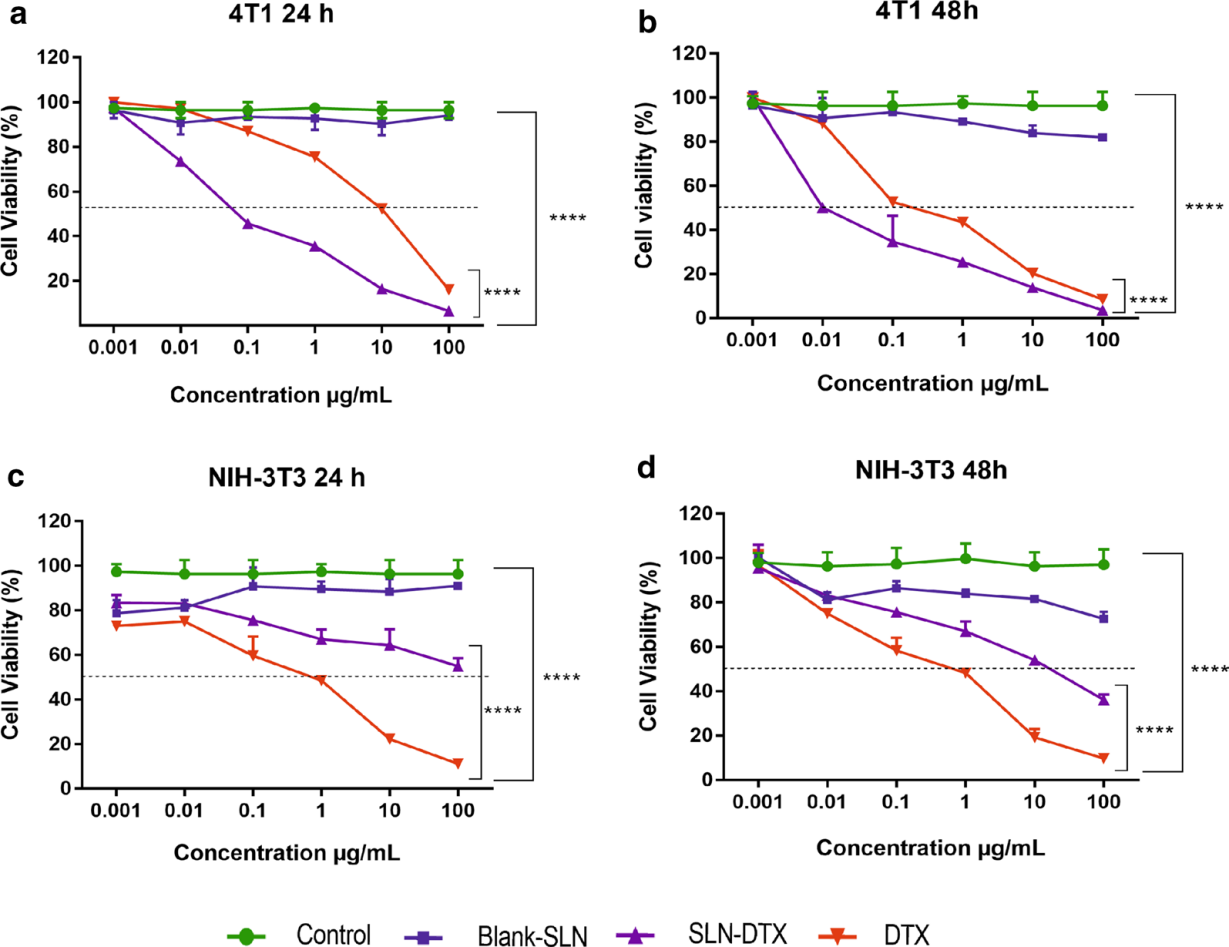
Viability of 4T1 (**a**, **b**) and NIH-3T3 (**c**, **d**) cells after 24 and 48 h treated with free DTX, blank-SLN and SLN-DTX at 0.001, 0.01, 0.1, 1, 10 and 100 µg/mL (equivalent DTX concentrations), evaluated by MTT assay. Data are expressed as mean ± standard error of the mean (****p < 0.0001)

**Fig. 5:**
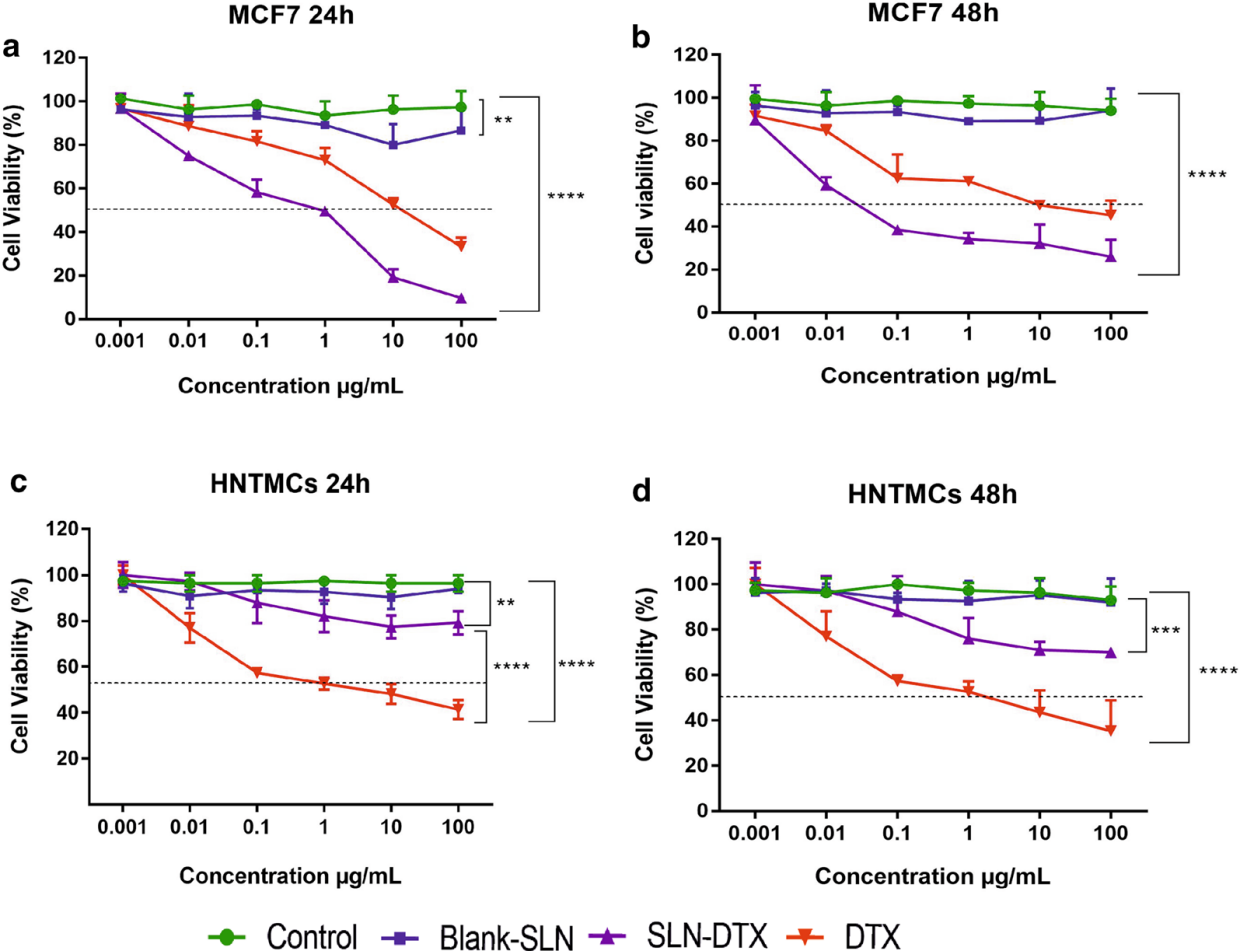
Viability of MCF7 (**a**, **b**) and HNTMCs (**c**, **d**) cells after 24 and 48 h treated with free DTX, blank-SLN and SLN-DTX at 0.001, 0.01, 0.1, 1, 10 and 100 µg/mL (equivalent DTX concentrations), evaluated by MTT assay. Data are expressed as mean ± standard error of the mean (**p < 0.01; ***p < 0.001; ****p < 0.0001)

To evaluate the cytotoxicity of SLN-DTX and free DTX, the MTT assay was performed using breast cancer cell lines (4T1 and MCF7) and non-cancerous cells (NIH-3T3 and HNTMCs). The results were compared with that of a clinically available DTX formulation, and the cytotoxicity of Blank-SLN was also investigated to exclude any non-specific effect. Cells were treated at 0.001, 0.01, 0.1, 1, 10 and 100 µg/mL equivalent DTX concentrations for 24 h (Figs. 4 and 5a, c) and 48 h (Figs. 4 and 5b, d). Blank-SLN had no effects on the cell viability and showed a similar result to the non-treated cells. It can be inferred that the blank-SLN composition is biocompatible and suitable for usage in intracellular applications.

The IC_50_ values of SLN-DTX against 4T1 cells were found to be 0.08 µg/mL (24 h) and 0.01 µg/mL (48 h) and for free DTX were 10 µg/mL (24 h) and 0.3 µg/mL (48 h), indicating time and dose-dependent cytotoxicity (Fig. 4a, b). The data of MCF7 cells demonstrate that the IC_50_ values of SLN-DTX were 1.04 µg/mL (24 h) and 0.05 µg/mL (48 h) and for free DTX were 12.19 µg/mL (24 h) and 10.01 µg/mL (48 h) (Fig. 5a, b). SLN-DTX showed higher cytotoxicity against cancer cells, while free DTX was toxic for both human and murine non-cancerous cells (HNTMCs and NIH-3T3). SLN-DTX demonstrated a significantly reduced IC_50_ value (p < 0.0001), which was found to be more than 100 times lower in concentration than free DTX, after 24 h of treatment, indicating higher cytotoxicity and efficiency of nanoparticles. Thus, it was concluded that the SLN-DTX was more effective, according to the release study, since a lower drug amount was released (69.4 µg/mL) in 24 h, compared to the free DTX that released 1000 µg/mL at the same time, and resulted in cytotoxic effects comparable. These results are in agreement with previous studies that showed that the cytotoxicity of drug-loaded lipid-based nanoparticles was higher than that of free drugs [4, 34, 47, 51, 52]. The enhanced efficacy mechanism of SLN-DTX cytotoxicity could be due to its rapid absorption and high permeability, which permit the intracellular nanoparticles to accumulate, mediated by endocytosis [2, 16], as shown in our TEM (internalization) and uptake results.

### 4T1 morphology

**Fig. 6:**
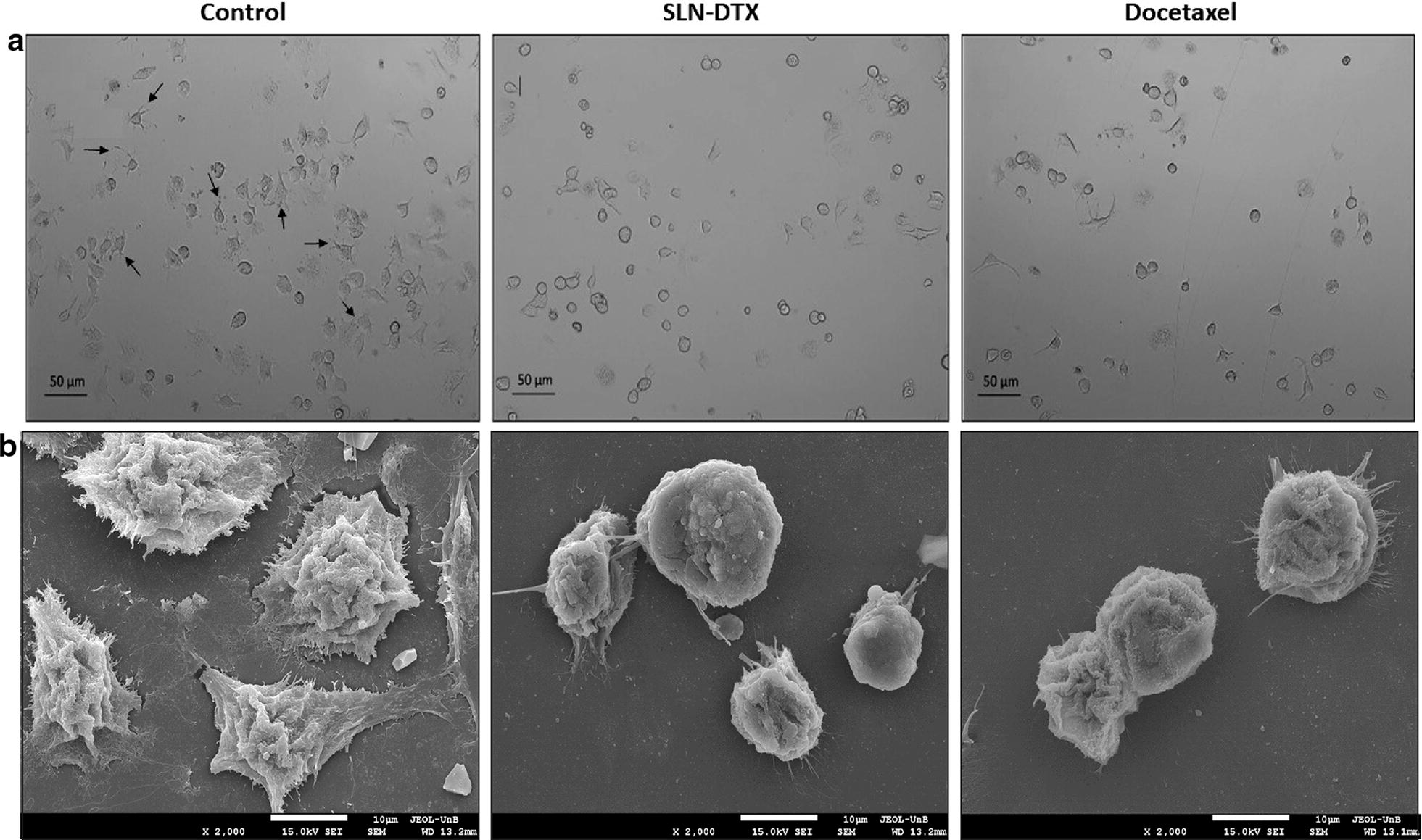
Morphology of murine breast adenocarcinoma cells (4T1). **a** Light microscopy (phase contrast). **b** Scanning electron micrograph (SEM). Control cells (without treatment), cells treated with SLN-DTX or DTX. The arrows show the cytoplasmic projection in cells. Cells were treated at 10 µg/mL for 24 h. Objective: 10×. The micrographs (SEM) were performed at 2 k

Phase contrast and scanning electron microscopy observations illustrated that the treated and untreated cells have morphological differences. These differences were observed with both techniques used (Fig. 6). The control cells showed a smooth cellular surface with characteristic cytoplasmic projections in most of them, as expected (Fig. 6a). The shape of both cells, after exposure with DTX and SLN-DTX treatments, was altered, the cytoplasmic projections decreased. Previous studies have shown similar results [53, 54]. These morphological surface alterations may be related to the disruption in the cytoskeleton dynamics promoted by DTX [55]. Impairment in the microtubule remodeling, induced by DTX, can affect cell surface morphology.

### Cell cycle analysis and cell apoptosis

**Fig. 7:**
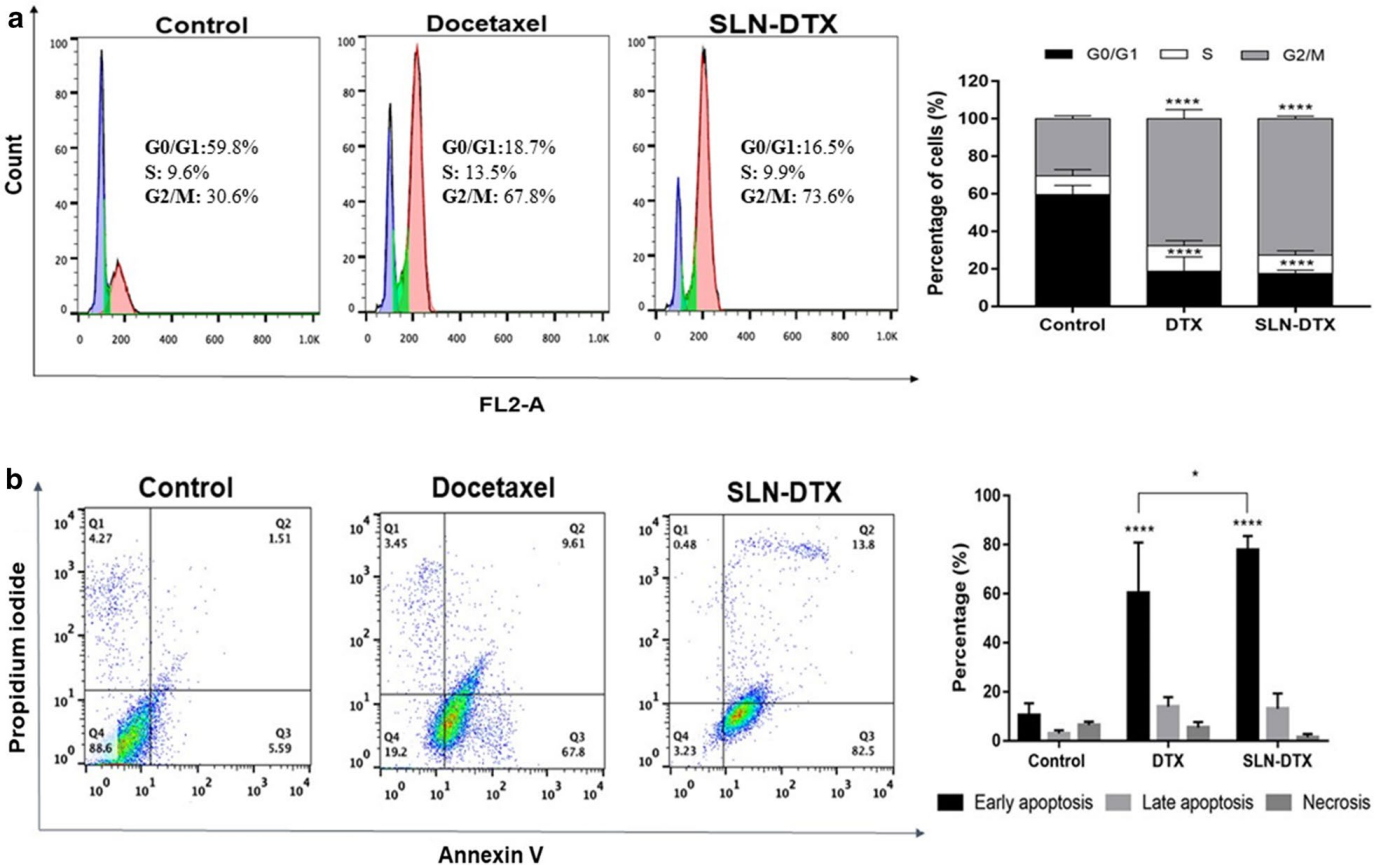
**a** Representative flow cytometry profiles of the cell cycle phase distribution of 4T1 cells. The first peak indicates 2n DNA content in the G0/G1 phase (purple), the second peak indicates 4n DNA content in the G2/M phase (pink), and in-between the two peaks is the S phase (green). **b** Dot plots representing the distribution of apoptotic cells after dual staining with Annexin V-FITC (horizontal axis) and propidium iodide (vertical axis). Annexin V-FITC and propidium iodide tagged cells symbolize early apoptosis (lower right quadrant) and late apoptosis (upper right quadrant), respectively. On the right side the percentage of the cells is presented. Groups: untreated cells, SLN-DTX and DTX. Cell were treated with 10 µg/mL for 24 h. Data are presented as mean ± standard error from three experiments (*p < 0.05; ****p < 0.0001)

Cell cycle deregulation is one of the hallmarks of cancer cells. Therefore, the induction of cell cycle arrest may be a good and effective strategy to control aberrant cancer cell proliferation [56]. Docetaxel has been shown to induce microtubule damage, which results in prolonged G2-M arrest and subsequent apoptosis [57]. Cell cycle analysis of 4T1 cells exposed to SLN-DTX or DTX is shown in Fig. 7-a. Consistent with previous results [57–59], cells treated with SLN-DTX or DTX resulted in an arrest in the phase of G2-M, with a significant decrease in G0/G1 phase versus control cells. The accumulation in G2-M phase was significantly higher in cells treated with SLN-DTX (73.7%) than in cells treated with free DTX (67.8%), suggesting that DTX was released into the cytosol, which caused cell cycle arrest in the G2-M phase. These results are consistent with the data obtained by MTT analysis and provide further evidence for potential clinical applications of SLN-DTX.

Apoptosis is considered the main cell death mechanism in response to taxanes. Apoptosis occurs when cells externalize a lipid found on the inner surface of the cell membrane, phosphatidylserine, which can be labeled with fluorochrome-conjugated annexin V. Generally, viable cells that have intact membranes do not interact with propidium iodide (PI), while membranes of dead cells are permeable to PI [60]. It is possible to differentiate viable cells (stained negative for both annexin V-FITC and propidium iodide), early apoptosis (stained positive for annexin V-FTIC and negative for PI), late apoptosis (stained positive for both annexin V-FITC and PI), and necrosis (stained positive for PI) [59]. Apoptotic cells show changes at the morphological and biochemical level, such as nuclear cytoplasmic condensation and chromatin aggregation. On the other hand, necrosis shows a passive consequence of gross injury to the cell with physiological consequences that are very different from apoptosis. In necrosis, the cell’s ability to maintain homeostasis is impaired [61]. Early apoptosis is represented by the cells accumulating in the lower right quadrant of the dot plot drawn between Annexin V-FITC and propidium iodide. As shown in Fig. 7b, the early and late apoptotic rates in the control group were 5.5% and 1.5%, respectively. Early apoptotic cells in the presence of SLN-DTX exhibited a significantly higher rate of 82.5%, as compared to the free DTX, at 67.8%, which could be attributed to the enhanced availability of DTX in SLNs. These results are consistent with the data of cell cycle analysis and suggest that SLN-DTX significantly improved DTX-dependent apoptosis in 4T1 cells in comparison to DTX.

### Effects of DTX on microtubules in 4T1 cells

**Fig. 8:**
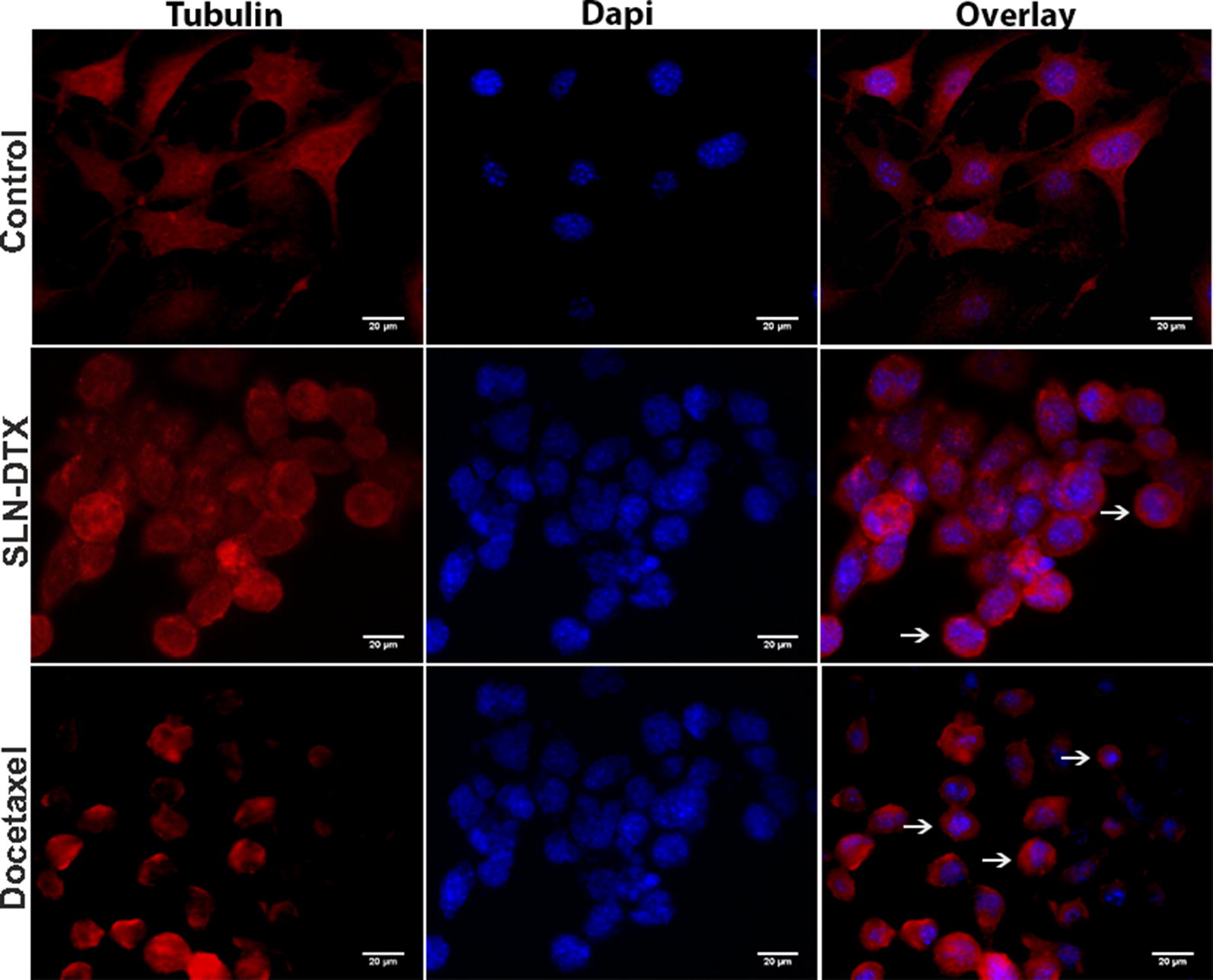
Immunofluorescence assay of 4T1 cells. Microtubules and nuclear staining are shown separately (Tubulin and DAPI, respectively) and combined (Overlay), for 4T1 cells untreated or treated with SLN-DTX or DTX 10 µg/mL for 24 h. The arrows show bundling of microtubules. (Objective 63×)

DTX has the ability to induce a cytotoxic effect by binding to the β tubulin subunit [62]. In Fig. 8, untreated cells are shown with clear, organized and extensive microtubule networks. However, cells treated with free DTX and SLN-DTX showed stabilized, shorter and straight microtubules resulting in elongated bundles of microtubules that curved around the cytoplasm, suggesting a change in the regulation of microtubule dynamics from DTX.

### Internalization of SLN-DTX by TEM and analysis of cellular uptake

**Fig. 9:**
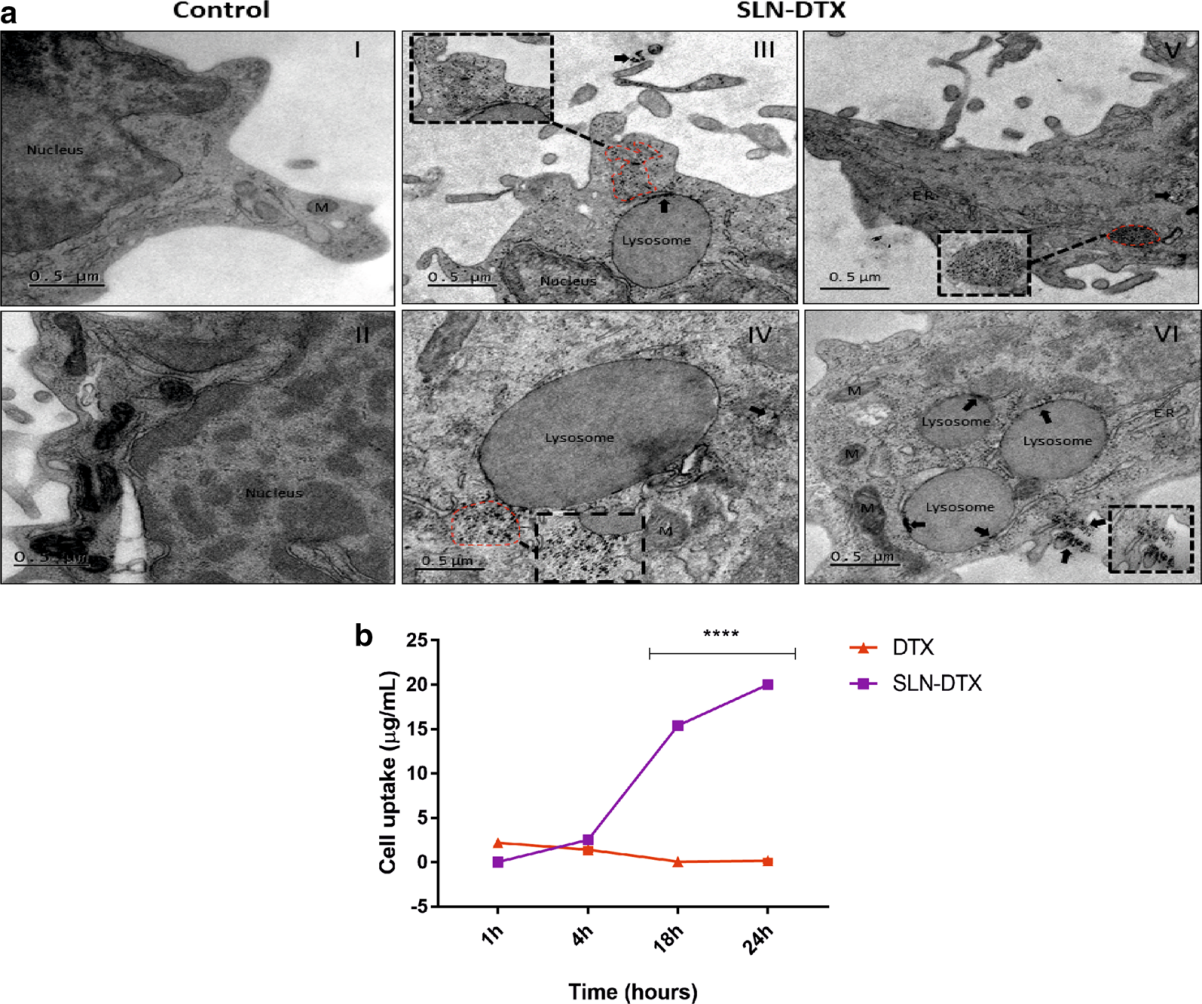
**a** Ultrastructural images of 4T1 cells. (I–II) Control cells (untreated). (III–VI) Cells treated with 10 µg/mL of SLN-DTX for 24 h. (III–V) SLN-DTX internalized (dashed line); (V) cluster of SLN-DTX in cytoplasm (dashed line); (VI) internalization of SLN-DTX into lysosomes (arrows). Dashed lines, arrows (into lysosomes) and arrow heads indicate nanoparticles. *ER* Endoplasmic reticulum, *M* Mitochondria. Magnification: 5 K; 15 K (black dashed lines). **b** In vitro cellular uptake studies of DTX in 4T1 cells. Cells were exposed to SLN-DTX or DTX for 1 h, 4 h, 18 h and 24 h at 100 µg/mL. Data are expressed as mean ± standard error of the mean (****p < 0.0001)

TEM was used to verify the cellular ultrastructure features and uptake of SLN-DTX. Lipid-based nanocarriers, such as SLNs, have been widely used as pharmaceutical carriers to increase the efficacy of chemotherapeutics [63]. Previous studies have demonstrated the internalization of lipid nanoparticles by endocytic pathway in various cell types [63, 64]. Consistent with the manner of internalization, the endocytosed nanoparticles were mainly accumulated within lysosomes/and or endosomes in the cytosol of the cell (Fig. 9a III–VI). Some of the nanoparticles appear to be digested within the vesicles. It appears that nanoparticles are arranged in clusters and individual particles can still be recognized (Fig. 9a III–VI). The nucleus can be identified, and the images consistently indicate that SLN-DTX were not present in the nucleus (Fig. 9a III). The control cells can be observed in Fig. 9a I–II.

To evaluate cellular uptake efficiency, in 4T1 cells, the DTX intracellular accumulation was analyzed by HPLC method. Cellular uptake was performed for SLN-DTX compared with free DTX. Results showed that DTX uptake from SLN-DTX by the cell was significantly higher at periods of 4 h, 18 h and 24 h than for free DTX. The DTX uptake accumulation is time-dependent, increasing over time, in cells treated with SLN-DTX. Otherwise, cells treated with free DTX have accumulation maintained up to 4 h and then DTX concentration decays (Fig. 9b). Mosallaei et al. [8] showed that TXT from the uptake of solid lipid nanoparticles in C-26 cells is greater when compared to the free TXT. These findings suggest that the use of SLN-DTX may allow doses of DTX to be decreased without loss of therapeutic effect, thereby reducing the drug’s toxicity.

### In vivo antitumor efficacy

**Fig. 10:**
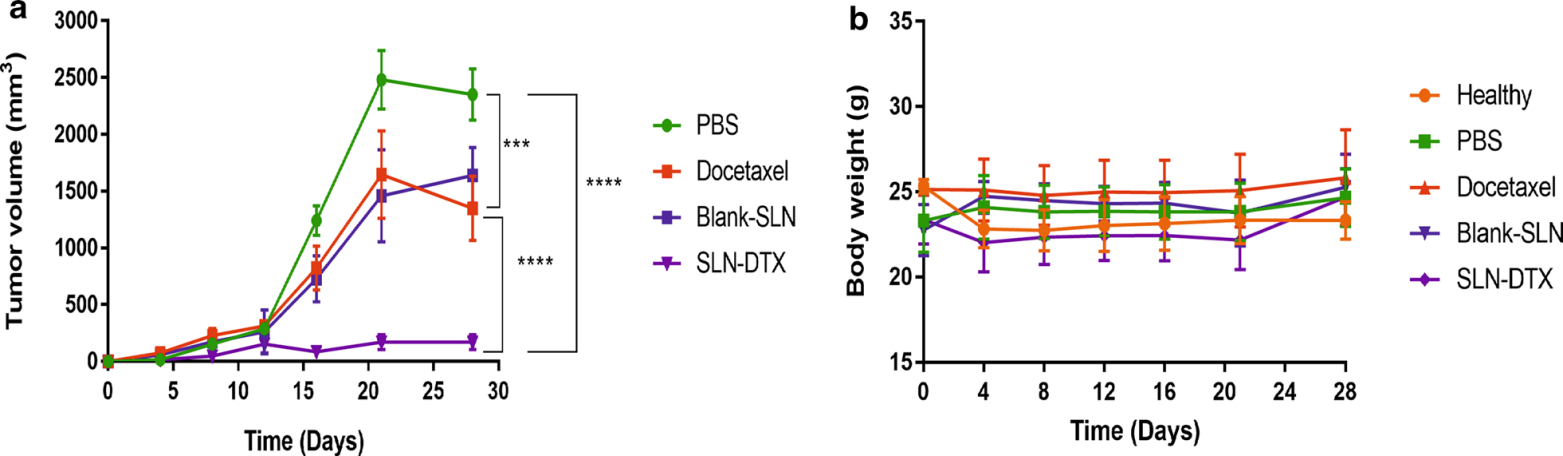
Antitumoral effect of SLN-DTX, Docetaxel, Blank-SLN and PBS on 4T1 tumor-bearing Balb/c mice. **a** Variation of tumor volume and **b** mice weight. ***p < 0.001; ****p < 0.0001, compared with PBS group

The in vivo therapeutic performances of SLN-DTX and free DTX were studied in 4T1 tumor-bearing Balb/c mice at 10 mg/kg DTX for a total of five doses. The dose was selected based on the previous works in literature [8, 44, 65]. In agreement with the in vitro cytotoxicity data, the group treated with SLN-DTX showed the lowest tumor growth rate (Fig. 10a). The results of tumor volume showed that the tumors of mice in the PBS and Blank-SLN groups grew rapidly, but the tumors in the docetaxel and SLN-DTX groups grew slowly. Treatments with docetaxel inhibited the tumor growth by about 42.7%, and SLN-DTX by 92.7% at the end point of study, which was significantly smaller than that in the PBS group. A previous report [16] showed tumor regression of mice treated with encapsulated DTX (10 mg/kg) was more effective than free DTX, which corroborated our findings.

There was no statistically significant variation in mice weight between the groups during the study, showing the negligible systemic toxicity of different treatments with the given dose (Fig. 10b). There were also no significant differences in the hematological and biochemical parameters (Additional file 6: Table S3).

### Lung metastasis prevention and IL-6 serum level of 4T1 breast cancer

We studied prevention of spontaneous metastasis in lung by SLN-DTX treatment. Spontaneous metastasis usually occurs in late stages of cancer, and the metastasis of 4T1 breast cancer occurs primarily in the lungs, sharing many characteristics with human breast cancer [66].

**Fig. 11:**
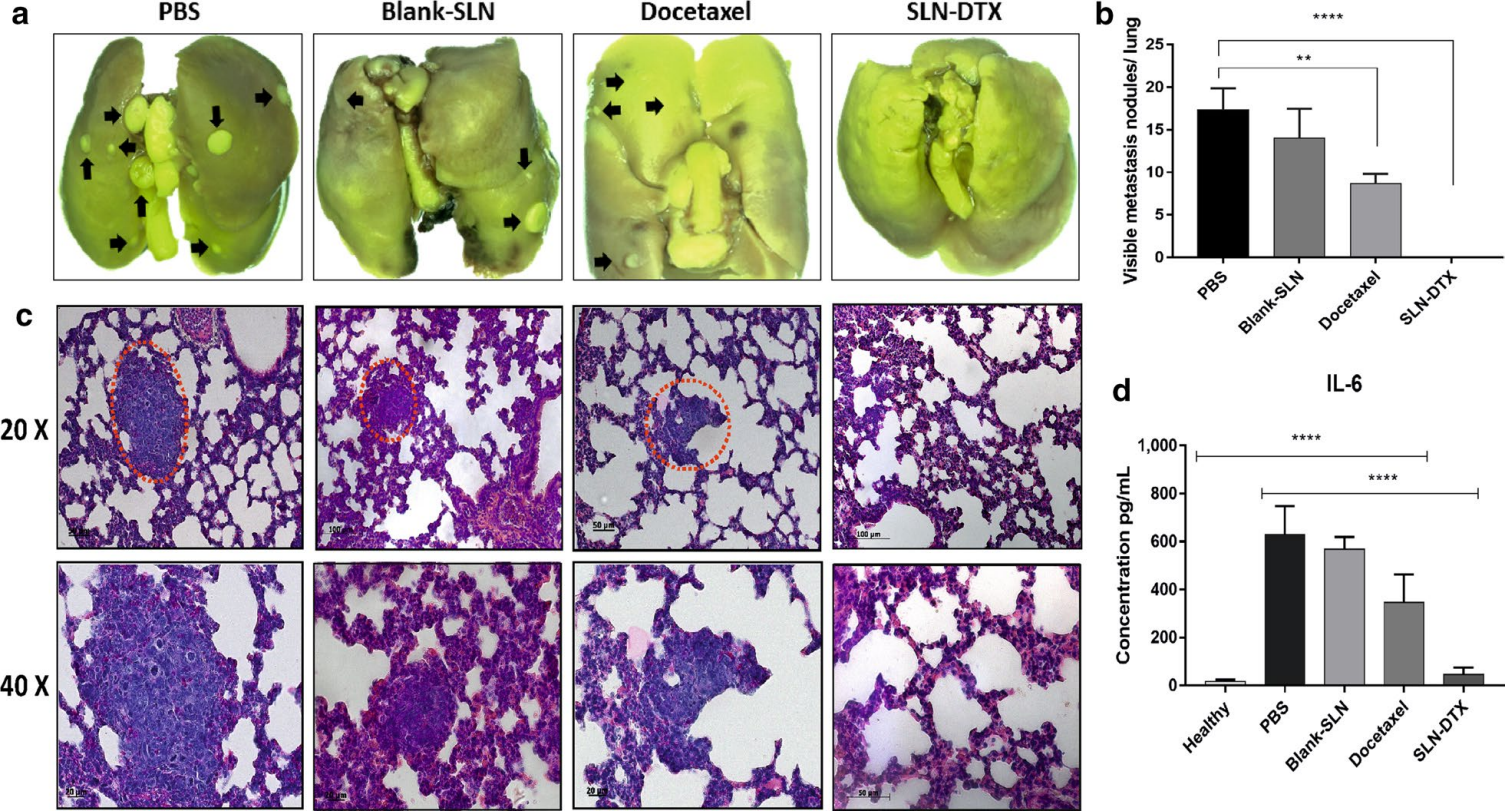
**a** Photo of lung. **b** Statistical analysis of numbers of tumor nodules on the lungs. **c** Histopathological examination of the lungs. **d** IL-6 serum levels of mice were measured by ELISA. The metastasis was marked by red dotted circle and black arrows. Data represent mean values ± standard error (**p < 0.01; ****p < 0.0001)

In PBS and Blank-SLN groups, we could clearly see many metastatic nodules about the lungs (Fig. 11a). After treatment with DTX, metastatic nodules were slightly reduced. In contrast, there was no metastasis in the SLN-DTX group, suggesting that SLN-DTX could significantly inhibit 4T1 breast cancer metastasis to the lung, also decreasing numbers of tumor nodules, and the size of tumor nodules (Fig. 11b). The metastasis was further confirmed by H&E staining analysis. As shown in Fig. 11c, many tumor metastasis loci (red dotted circle) were observed in lungs of PBS and Blank-SLN groups and a moderate number in lungs of docetaxel group, while there was no metastasis in the lungs of SLN-DTX group. These results suggest that SLN-DTX has a greater ability to inhibit tumor metastasis to lungs than free docetaxel.

Unbalanced cell proliferation is correlated with tumor progression, evasion from immune response and metastatic potential. These changes are associated with alteration in the production of cytokines [67]. Interleukin-6 (IL-6) is a pleiotropic cytokine that functions in the regulation of pro-inflammatory and metastatic tumor. Studies have shown increased circulating levels of IL-6 in patients with breast cancer. High IL-6 levels are also associated with cancer development, progression and metastasis [67, 68]. In addition, a previous study reported that breast tumor-bearing mice expressing high levels of IL-6 had spontaneous lung and liver metastasis [69]. Therefore, we evaluated IL-6 serum levels in mice after treatments.

In our results, IL-6 levels were significantly higher in groups with presence of metastatic disease in lungs (as shown in H&E and lung metastasis results) that is the PBS, Blank-SLN and DTX (628 ± 3.4; 568 ± 1.7; 345 ± 6.6 pg/mL; p < 0.0001) groups, when compared with the healthy control group. There was no significant difference between the healthy control group and the SLN-DTX treated group (Fig. 11d). These findings support the important role of IL-6 levels in breast cancer invasion and metastasis. In studies carried out by Salgado et al. [67] high IL-6 serum levels were shown to be associated with lower survival in patients with advanced breast cancer and, at later stages, high levels may stimulate tumor growth.

### Bcl-2 and Ki-67 immunohistochemistry analysis

**Fig. 12:**
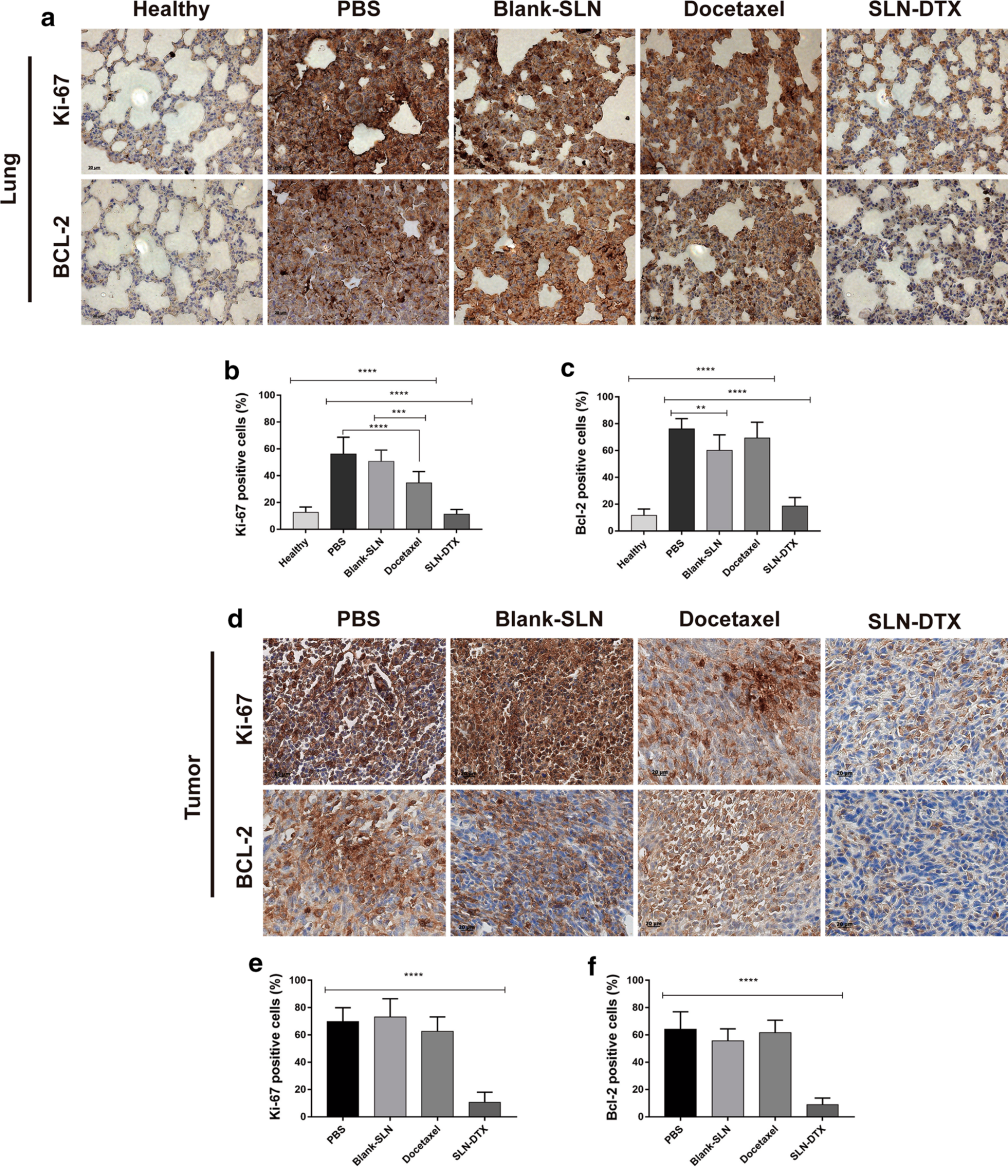
Immunohistochemistry analysis. The brown dye-stained cells represent positive cells. **a** Lung and **d** tumor slides were immunostained with anti-Ki-67 and BCL-2. **b**, **e** Quantification of Ki-67 and **c**, **f** BCL-2 staining. Data represent mean values ± standard error (**p < 0.01; ***p < 0.001; ****p < 0.0001)

Bcl-2 is a proto-oncogene related with programmed cell death/apoptosis. Studies have shown the overexpression of Bcl-2 protein increase invasion and migration in breast cancer [70, 71]. In this context, based on results we found regarding metastasis prevention by SLN-DTX treatment, we investigated Bcl-2 in lungs and tumor tissues by immunohistochemistry (Fig. 12). Strong staining of Bcl-2 was observed in groups previously identified with pulmonary metastasis, that is, the PBS, Blank-SLN and DTX groups, when compared to SLN-DTX group, which showed statistically significant differences (Fig. 12c, f) (p**** < 0.0001).

We performed Ki-67 to examine cell proliferation in the tumors and lungs after treatments and control groups. The analysis revealed high positive indexes of PBS, Blank-SLN and DTX groups. The SLN-DTX treatment induces significantly lower (****p < 0.0001) levels of cellular proliferation in comparison with control PBS group (Fig. 12b, e). Therefore, these results suggested that SLN-DTX induced the reduction of cell proliferation and that it is associated with tumor growth suppression, as observed previously.

## Conclusions

SLN-DTX was successfully prepared using the high-energy method. The results showed that SLN-DTX is stable over time, with uniform distribution and high encapsulation efficiency (86%). The in vitro release study showed a sustained and continuous release pattern of SLN-DTX. The amorphous state of DTX in SLN-DTX was confirmed by DSC. The in vitro tests proved that SLN-DTX has higher cytotoxicity against tumor cells, an efficient cellular uptake and arrested cell cycle progression in the G2/M stage; it also induced more apoptosis in 4T1 cells at a lower dose compared with DTX. SLN-DTX was taken up by the 4T1 cells through an endocytosis process, localized mostly in the lysosomes. In vivo study indicated that SLN-DTX not only significantly inhibited the tumor growth of 4T1 breast cancer, but also prevented lung metastasis at a dose of 10 mg/kg that did not cause noticeable systemic toxicity in mice. Our findings suggest that SLN-DTX inhibits tumor growth and prevents lung metastasis by reducing the production of IL-6 serum level, inducing apoptosis of the tumor cells and reduction of tumor cell proliferation and BCL-2 expression. In conclusion, these results endorse the proposal that SLN-DTX can be considered as an alternative and promising biocompatible formulation to be used for the treatment of breast cancer.

## Materials

Docetaxel, MTT, Annexin, Propidium iodide, Pluronic F127, Span 80 and Compritol ATO 888 were purchased from Sigma. Mouse mammary cell line (4T1), human breast cancer cells (MCF7) and mouse embryo fibroblast cell line (NIH-3T3) were acquired from the Rio de Janeiro Cell Bank. Dulbecco’s modified Eagle’s medium (DMEM), Roswell Park Memorial Institute medium 1640 (RPMI) medium, fetal bovine serum (FBS), penicillin and streptomycin were bought from Gibco, Invitrogen. MTT (3-[4,5-dimethylthiazol-2-yl]-2,5 diphenyltetrazoliumbromide) came from Thermo Fisher; osmium tetroxide from Electron Microscopy Sciences, Hatfield, PA; and acetonitrile (99.95%—grade HPLC) from J.T. Backer. All other chemicals were of analytical grade.

## Methods

### Preparation of solid lipid nanoparticles (SLNs)

Blank-SLN (without docetaxel) or SLN-DTX were prepared by the high-energy method. In brief, aqueous and oil phases were prepared separately. The oil phase of Compritol (500 mg), Span 80 (50 mg) and Pluronic F127 (450 mg) was melted and kept at 80 °C and then DTX (20 mg) was added to oil phase until it melted completely. The aqueous phase (20 mL of PBS) was added dropwise to the oil phase at the same temperature (80 °C) by continuous high-speed stirring using Ultra-Turrax T25 (IKA-Werke, Saufen, Germany) at 10,000 rpm for 10 min. The final status of the products, is it dispersed in PBS.

### Determination of hydrodynamic diameter (HD), polydispersity index (PDI) and zeta potential

The physicochemical parameters including hydrodynamic diameter, PDI and zeta potential of SLN-DTX were recorded over the period of 120 days by dynamic light scattering (DLS) using Zeta Sizer (Nano ZS, Malvern Instruments, UK) at 25 °C. All measurements were carried out after dilution (1:20) with distilled water.

In order to evaluate the storage stability, SLN-DTX was stored at 4 °C, and after 24 h was withdrawn and analyzed for mean hydrodynamic diameter, PDI and zeta potential by DLS. Then, the same sample was stored at 37 °C for 24 h and the measurement was performed, so on until completing 5 total readings. The same parameters were measured after the end of each cycle.

Interaction between SLN-DTX and biological media were performed with sample dilution (1:20) in distilled water, PBS, cell media (10% v/v in PBS) and serum (10% v/v in PBS). DLS was used to record hydrodynamic diameter, PDI and zeta potential up to 7 days after formulation.

### Transmission electron microscopy

The size and morphology of SLN-DTX were characterized using transmission electron microscope—TEM (JEOL 1011, Tokyo, Japan) operated at accelerating voltage of 100 kV. Samples were prepared by placing a drop of sample (5 µL) using a 400-mesh copper grid coated with Formvar film, and then it was air-dried, following contrast enhancement with osmium tetroxide vapor. Grids were kept at room temperature until analysis. A minimum of 400 particles were measured using ImageJ software.

### Quantification of DTX by high performance liquid chromatography (HPLC)

DTX concentration was analyzed by HPLC (Shimadzu, Kyoto, Japan). The stationary phase consisted of a reversed phase ACE AQ C18 column (25 × 0.4 cm, 5 µm particle diameter) (ACE Aberdeen, Scotland) with a precolumn (1 × 0.4 cm, 5 µm particle diameter) (ACE Aberdeen, Scotland). The column temperature was maintained at room temperature. The mobile phase consisted of acetonitrile/deionized water (55:45, v/v).

The injection volume was 20 µL, the flow rate was 1 mL/min at a pressure of 120 kgf/cm^−2^. The temperature of the column was maintained at 30 °C during all the runs. UV detection was performed at a wavelength of 232 nm. A calibration curve (y = 21752x − 128538, R^2^ = 0.9994) was drawn using different concentrations of DTX with a range of 0.01–1 mg/mL.

### In vitro drug release study

The determination of the drug release behavior from SLN-DTX was performed by the dialysis bag (14 kDa Sigma, USA) method. Phosphate-buffer saline (PBS, pH 7.4 or pH 5.0) containing 0.1% Tween-80 was used as the release medium. The bag containing SLN-DTX (1 mg/mL of DTX) was incubated in 50 mL release medium at 37 ± 0.5 °C under horizontal shaking speed of 100 rpm. Samples at specified time intervals (0, 0.25, 0.5, 1, 2, 4, 6, 24, 48, 72, 120 and 240 h) were withdrawn and replaced immediately with the same volume of fresh release medium. The released DTX content was determined by HPLC.

### Determination of encapsulation efficiency and drug loading of SLN-DTX

SLN-DTX (formulation dispersed in PBS) in was submitted to centrifugation at 3000 rpm for 10 min and then the supernatant was collected, diluted 1:10 (v/v) in DMSO and submitted to the ultrasonic bath for 5 min to promote separation of the DTX from the others lipid components. This mixture was then diluted (1:100, v/v) in the mobile phase that consisted of acetonitrile and deionized water with a ratio of 55:45 (v/v), submitted to gentle centrifugation of 2500 rpm at 5 min, to promote sedimentation of residual lipids, then analysis by HPLC under the conditions mentioned in the above item (Quantification of DTX by high performance liquid chromatography (HPLC)). The entrapment efficiency (EE%) and drug loading (DL%) were calculated by the following formulas:

1 $$Entrapment\;efficiency\;({\text{EE}\%}) = \frac{weight\;of\;DTX\;in\;SLNs}{weight\;of\;DTX\;used\;in\,SLNs\; preparation} \times 100$$

2 $$Drug\;loading\;({\text{DL\%}}) = \frac{weight\;of\;DTX\;in\;SLNs}{Weight\;of\;SLNs} \times 100$$

### Fourier transform infrared spectroscopy (FTIR)

The changes within the functional groups of the samples were observed using Fourier transform-infrared spectroscopy (Vertex 70, Bruker, Billerica, USA). FTIR spectrum of DTX, Compritol ATO 888, Span 80, Pluronic F127, SLN-DTX and Blank-SLN were obtained by ATR (attenuated total reflectance) method. FTIR spectra were measured over the range of 4000–400 cm^−1^ with resolution of at 4 cm^−1^ for 50 scans.

### Differential scanning calorimetry (DSC)

Thermograms of Compritol ATO 888, Span 80, Pluronic F127, DTX, SLN-DTX and Blank-SLN were obtained by using differential scanning calorimetry DSC-60 (Shimadzu, Kyoto, Japan). DTX in dry powder format and the other samples in liquid form. Samples were weighed, a mass of 4.4 mg, 22 mg, 12.3 mg, 3.2 mg, 2.6 mg, 9.5 mg of Compritol ATO 888, Span 80, Pluronic F127, DTX, SLN-DTX and Blank-SLN, respectively, in DSC aluminum crimped pans, and an empty pan was used as reference. DSC was performed at 25–400 °C temperature range at the rate of 10 °C/min under N_2_ flow to provide inert atmosphere during the measurement to prevent oxidation reaction.

### Confocal raman microscopy (CRM)

SLN-DTX, Blank-SLN, Compritol ATO 888, Span 80 and Pluronic F127 spectroscopic analyses were performed by a confocal Raman spectrometer (Witec GmbH, Germany) with a 532 nm laser at 30 mW. The spectra were recorded at an integration time of 0.15 s per point.

### Cell culture

Murine breast adenocarcinoma cells (4T1), human breast cancer cells (MCF7) and murine embryo fibroblast (NIH-3T3) cells were cultured in RPMI-1640 or DMEM, supplemented with 10% fetal bovine serum (v:v), penicillin (100 U/mL), and streptomycin (100 U/mL), at 37 °C in a humidified atmosphere containing 5% CO_2_. Primary cultures of Human non-tumor mesenchymal cells (HNTMCs), provided informed consent and with approval from the human ethics committee of the University of Brasilia (104934/2008). HNTMCs were cultured in DMEM. Cells in the logarithmic growth phase were used in the following cell experiments. NIH3-3T3 and HNTMCs were used as non-tumor control cells.

### Cell viability assay

The cytotoxicity was determined by colorimetric-based MTT assay. Cells were seeded in a 96-well plate at a density of 4 × 10^3^ cells/well and incubated overnight. Then, each well was replaced with fresh medium containing different doses of DTX, Blank-SLN, SLN-DTX, or culture medium (negative control) for 24 or 48 h culture. Subsequently, the culture medium was replaced with one containing MTT (5 mg/mL), and cells were incubated for 4 h at 37 °C. Then, the supernatant was dropped and DMSO was added to each well to dissolve the formazan crystals. Absorbance was measured at 595 nm. Untreated cells were taken as control with 100% viability. The drug concentration at which the growth of 50% cells was inhibited (IC_50_) in comparison with that of the control sample was calculated by curve fitting of the cell viability data. All experiments were performed in triplicate.

### Cell morphology

The morphology and confluence of 4T1 cells were analyzed using a phase contrast microscope (Zeiss, Germany) and the AxioVision^®^ software (Zeiss, Germany).

Twenty-four hours after treatment (10 µg/mL), 4T1 cells were fixed using Karnovsky overnight. Samples were washed with cacodylate buffer (0,1 M), post-fixed with osmium tetroxide and dehydrated using graded acetone (30–100%). Then, they were critical-point-dried (Balzers, CPD 030, Germany) from liquid CO_2_ and gold-sputtered. The cell morphology was observed using scanning electron microscopy.

### Cell cycle

Cell cycle analysis was carried out by staining the DNA at different stages with propidium iodide (PI), determined by flow cytometry. Briefly, 1 × 10^5^ cell/well were seeded in 24-well cell culture plates. The 4T1 cells were exposed to SLN-DTX or DTX treatment (10 µg/mL) for 24 h. Cells were washed with PBS and trypsinized, then harvested and centrifuged. The pellet was washed in PBS, fixed in ice-cold ethanol (70%) at 4 °C. Then, cells were stained with propidium iodide solution consisting of 45 mg/mL PI, 10 mg/mL RNase A, and 0.1% Triton X-100. After 1 h of incubation at 37 °C in a CO_2_ incubator, flow cytometry analysis was performed using FACSCalibur (BD Biosciences Co., Franklin Lakes, New Jersey, USA). The data were analyzed using FlowJo single cell analysis software.

### Cell apoptosis analysis

Annexin-V staining was performed to differentiate apoptosis from necrotic cell death induced by SLN-DTX or DTX. The same dose that had been applied to the cells and the same incubation time as in cell cycle analysis were used. The cells were then harvested, washed with cold PBS and centrifuged. The cell pellet was resuspended in annexin-binding buffer, and then Annexin V FITC conjugate and propidium iodide solution were sequentially added to the sample in order to detect apoptotic and necrotic cell populations, respectively. Finally, the cells were incubated at room temperature for 15 min and measurement was conducted by flow cytometer. A total of 20,000 events were collected per sample.

### Immunocytochemical staining

4T1 cells were cultured in twelve-well plates with cover glass for 24 h and then treated with SLN-DTX or DTX (10 µg/mL). Normal culture medium was used as a control. After 24 h of incubation, the cells were fixed with 4% paraformaldehyde for 15 min. The cells were washed with PBS and permeabilized with 0.5% Triton X-100 for 10 min. The cells were stained with a primary antibody against tubulin at a dilution of 1:500 at 4 °C overnight. A FITC-conjugated goat anti-rabbit IgG was used to combine the tubulin antibody. The nucleus was stained with DAPI (0.1 µg/mL) for 7 min and the cover slips were mounted on glass slides. The cells were analyzed with a Zeiss Axiophot microscope (Carl Zeiss, Jena, Germany).

### In vitro cellular uptake

The cellular uptake of DTX by 4T1 cells was analyzed quantitatively, performed by analytical method of HPLC. The cells (10^5^ cells/well) were seeded in 24-well plates. Cells were treated with SLN-DTX or DTX (100 µg/mL) for 1, 4, 18, and 24 h at 37 °C under 5% CO_2_. At the end of the incubation period, cells were washed three times with ice-cooled PBS to remove any SLN-DTX or DTX that remained in the surrounding matrix or on the cell membrane surface. Cells were trypsinized for 15 min until all the cells were detached; 800 µL methanol was added to each well and it was shaken for 3 min. After that, each sample was kept at 4 °C for 48 h to ensure the destruction of nanoparticle structure and dissolving of all DTX in methanol. Cells were vortexed for 1 min, and centrifuged for 10 min at 1000g at 4 °C. The supernatant was kept and analyzed by HPLC method in the same way as mentioned previously in “Quantification of DTX by high performance liquid chromatography (HPLC)” section.

### Internalization of SLN-DTX in 4T1 cells

Briefly, 4T1 cells were seeded (1 × 10^6^) and treated with SLN-DTX (10 µg/mL) for 24 h. Then, cells were fixed with Karnovsky fixative and post-fixed with 1% osmium tetroxide containing potassium ferricyanide for 1 h at room temperature. Samples were dehydrated in grading acetone (30–100%) and embedded in Epon resin. Ultrathin sections were stained with 5% uranyl acetate. Ultrastructural studies were performed using a transmission electron microscope (JEOL 1011, Tokyo, Japan) at accelerating voltage 80 kV.

### Evaluation of the antitumor activity of the SLN-DTX in vivo

Balb/c female mice (12 weeks old) were purchased from the Catholic University of Brasilia (Brasília, Brazil). All experiments described were approved by the Animal Research Ethics Committee of the University of Brasilia, Brazil. 4T1 cells (4 × 10^5^ cells per mouse) were subcutaneously injected into the left flank. The tumors were allowed to grow for 2 weeks. The mice were randomized into five groups with 6 mice in each group; healthy (without tumor), phosphate buffered saline (PBS), Blank-SLN, free DTX and SLN-DTX at a fixed dose of 10 mg/kg. The animals were treated intraperitoneally every 4 days, totalizing five applications. The body weights of mice were monitored as an index of systemic toxicity [72]. The tumor size was measured using a digital caliper and calculated using the following formula 4π/3 × (length/2) × (width/2)^2^ [51]. On day 28, mice were euthanized, and tumors and lungs were removed. Lungs were fixed in Bouin’s solution and metastatic nodules were counted.

### Hematology and blood chemistry tests

Blood samples were collected to carry out hemogram and biochemical dosages. Hemogram was processed in a multiple automated hematology analyzer (XZ 2100 Sysmex equipment) and serum biochemical analyses were run on the automated chemistry analyzer (ADVIA 2400, Siemens).

### ELISA

The cytokine IL-6 level was evaluated with an ELISA kit (Sigma, USA) according to the manufacturer’s protocol. Briefly, 100 µl of standard and mice plasma in duplicated wells were incubated at room temperature for 45 min. After washing, 100 μl of enzyme conjugate reagent was added into each well, then incubated. The reaction was stopped and samples were measured with a microplate reader.

### Histopathological and immunohistochemical evaluations

The histopathology of tumor and lung sections was evaluated by hematoxylin and eosin (H&E) method. Immunohistochemical evaluation was carried out in Micra laboratory (Brasília, Brazil). Tissues were embedded in paraffin, sectioned, and stained with an antibody against BCL-2 and Ki-67 (Abcam, Cambridge, MA, USA). Quantification was done in a blind fashion by counting positive cells in 10 fields (400×) using a light microscope (Zeiss, Germany).

Immunohis-tochemical staining was performed according to the protocol provided by the manufacturer.

### Statistical analysis

Statistical evaluation of data was performed using t-test, and one or two-way ANOVA analyses of variance, followed by Tukey’s post-tests using the GraphPad Prism 5.0 software. p < 0.05 was considered as the statistically significant difference. Quantitative results were expressed as mean ± standard deviation (SD).

## Supplementary information

**Additional file 1: Figure S1**. Colloidal stability of SLN-DTX in water, PBS, cell culture media and serum over 7 days. (A) Hydrodynamic diameter (HD), (B) Zeta potential and (C) Polydispersity index (PDI) measured by dynamic light scattering (p > *0.1).

**Additional file 2: Table S1.** Assignment of FTIR spectrum of Compritol, Pluronic, Span 80 docetaxel, solid lipid nanoparticles (Blank-SLN) and docetaxel-loaded solid lipid nanoparticles (SLN-DTX).

**Additional file 3: Figure S2.** FTIR spectra of docetaxel. The FTIR spectrum of DTX was obtained by ATR (attenuated total reflectance) method.

**Additional file 4: Figure S3.** Raman spectra of docetaxel. The Raman spectral signature of our Docetaxel was performed by a confocal Raman spectrometer with a 532 nm laser.

**Additional file 5: Table S2**. Raman vibrational wavenumbers (in cm^−1^) and approximate assignments of docetaxel and SLN-DTX.

**Additional file 6: Table S3.** Hematology and biochemical parameters of female Balb/c mice. Systemic toxicity assessment after DTX and SLN-DTX treatments on hematology and biochemical parameters of female mice 30 days after 4T1 cells implantation.

## Data Availability

The datasets used and/or analyzed during the current study are available from the corresponding author on reasonable request.
