# Supplementary figures and images for: Docetaxel-loaded solid lipid nanoparticles prevent tumor growth and lung metastasis of 4T1 murine mammary carcinoma cells
Source: J Nanobiotechnology. 2020 Mar 12;18:43. doi: 10.1186/s12951-020-00604-7 (PMC7068958; doi:10.1186/s12951-020-00604-7)

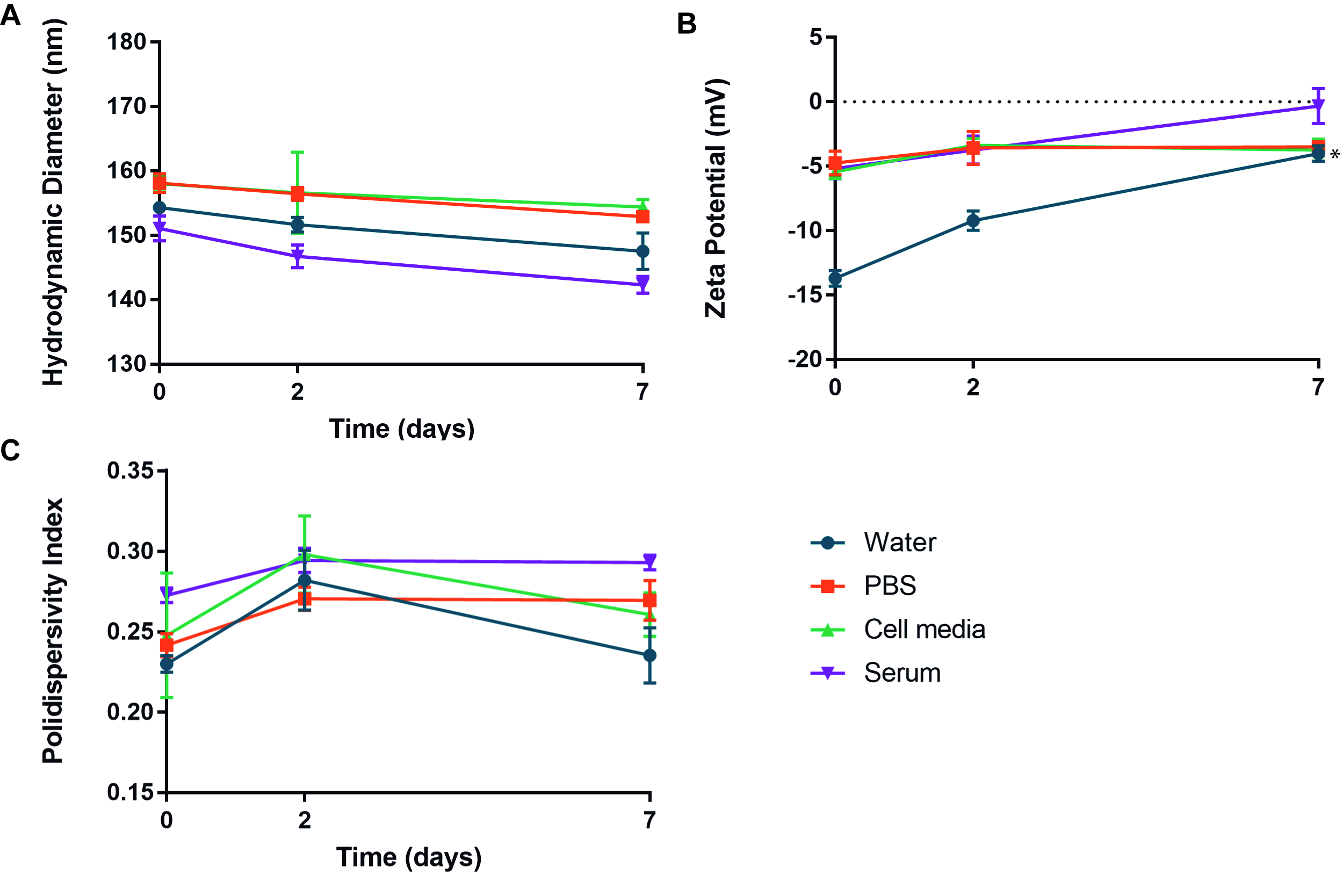

Supplement: Supplementary file 1 — Additional file 1: Figure S1. Colloidal stability of SLN-DTX in water, PBS, cell culture media and serum over 7 days. (A) Hydrodynamic diameter (HD), (B) Zeta potential and (C) Polydispersity index (PDI) measured by dynamic light scattering (p > *0.1). [file 12951_2020_604_MOESM1_ESM.tif]
