# Supplementary material for: Docetaxel-loaded solid lipid nanoparticles prevent tumor growth and lung metastasis of 4T1 murine mammary carcinoma cells
Source: J Nanobiotechnology. 2020 Mar 12;18:43. doi: 10.1186/s12951-020-00604-7 (PMC7068958; doi:10.1186/s12951-020-00604-7)
Supplement: Supplementary file 2 — Additional file 2: Table S1. Assignment of FTIR spectrum of Compritol, Pluronic, Span 80 docetaxel, solid lipid nanoparticles (Blank-SLN) and docetaxel-loaded solid lipid nanoparticles (SLN-DTX). [file 12951_2020_604_MOESM2_ESM.docx]

**Table S1** - Assignment of FTIR spectrum of Compritol, Pluronic, Span 80 docetaxel, solid lipid nanoparticles (blank-SLN) and docetaxel-loaded solid lipid nanoparticles (SLN-DTX).

| **Infrared peaks (cm^-1^)** | | | | | | **Functional groups** |
| --- | --- | --- | --- | --- | --- | --- |
| **Compritol** [27] | **Pluronic** [25] | **Span 80** [28] | **Docetaxel** [29] | **Blank-SLN** | **SLN-DTX** |  |
| 3423 |  | 3421 | 3460 | 3371 |  | νOH |
|  |  |  | 3369 |  |  | νNH |
|  |  |  |  |  | 3389 | νOH + νNH |
| 2955,2914 | 2967 | 2922 | 2981, 2938 | 2955,2916 | 2955,2916 | ν_as_CH |
| 2849 | 2880 | 2852 | 2853 | 2849 | 2849 | ν_s_CH |
| 1736 |  | 1740 | 1738, 1709 | 1736 | 1736 | νC=O |
|  |  |  | 1587 |  |  | δN-H |
|  |  |  | 1497, 1437 |  |  | νC=C (aromatic) |
| 1468 | 1466 | 1461,1377 | 1375, 1267, 1244, 1171, 1117, 1070, 1022 | 1466 | 1466 | δCH (CH_2_ alkane) |
|  |  |  |  | 1342 | 1342 | νC-O + νC-N |
|  |  |  |  |  |  | νCOC |
| 1190,1173 | 1342,1279 | 1169,1084 |  |  |  | νCO |
| 1109 |  |  |  | 1107,1060 | 1107 | νCO |
|  | 961, 841 |  | 974, 708 | 962 | 962 | δCH |
| 719 |  | 721 |  | 719 | 719 | *Rocking* CH_2_ |
