# Supplementary material for: Docetaxel-loaded solid lipid nanoparticles prevent tumor growth and lung metastasis of 4T1 murine mammary carcinoma cells
Source: J Nanobiotechnology. 2020 Mar 12;18:43. doi: 10.1186/s12951-020-00604-7 (PMC7068958; doi:10.1186/s12951-020-00604-7)
Supplement: Supplementary file 5 — Additional file 5: Table S2. Raman vibrational wavenumbers (in cm−1) and approximate assignments of docetaxel and SLN-DTX. [file 12951_2020_604_MOESM5_ESM.docx]

**Table S2**- Raman vibrational wavenumbers (in cm^-1^) and approximate assignments of docetaxel and SLN-DTX.

| **Samples (cm^-1^)** | | **Vibrational**  **assignment** |
| --- | --- | --- |
| **Docetaxel (DTX)** | **SLN-DTX** |  |
| 3074 | 3072 | ν(O-H) |
| 2983 | - | ν (CH_2_ e CH_3_) |
| 2937 | 2940 | ν (CH_2_ e CH_3_) |
| 2854 | - | ν (CH_2_ e CH_3_)) |
| 1715 | 1714 | νC=O |
| 1632 | 1636 | νC=C (aromtic) conjugated with C=O |
| 1605 | 1602 | νC=C (aromtic) + νCO |
| 1455 | 1465, 1442 | δCH (CH_2_ e CH_3_) |
| 1367 | 1369 | δCH (CH_2_ e CH_3_) |
| 1316 | - | δCH (CH_2_ e CH_3_) |
| 1275 | - | δC-alkyl |
| 1256 | - | νC-O (alcohol) |
| 1195, 1164, 1035, 1006 | 1167, 1004 | νC=C (aromatic) |
| 956 | - | Rocking CH (methyl) |
| 896 | 893 | Rocking CH methyl) |
| 799, 780, 763 | - | δCH |
| 694, 620 | - | νC=C (aromatic) |
