# Supplementary material for: Docetaxel-loaded solid lipid nanoparticles prevent tumor growth and lung metastasis of 4T1 murine mammary carcinoma cells
Source: J Nanobiotechnology. 2020 Mar 12;18:43. doi: 10.1186/s12951-020-00604-7 (PMC7068958; doi:10.1186/s12951-020-00604-7)
Supplement: Supplementary file 6 — Additional file 6: Table S3. Hematology and biochemical parameters of female Balb/c mice. Systemic toxicity assessment after DTX and SLN-DTX treatments on hematology and biochemical parameters of female mice 30 days after 4T1 cells implantation. [file 12951_2020_604_MOESM6_ESM.docx]

**Table S3-** Hematology and biochemical parameters of female Balb/c mice.

| **Parameters** | **Healthly** | **PBS** | **Blank-SLN** | **Docetaxel** | **SLN-DTX** |
| --- | --- | --- | --- | --- | --- |
| RBC mm^3^ | 6.6±2.5 | 7,1±1.9 | 6,5±1.6 | 6,5±2.7 | 6,6±2.2 |
| HGB g/dL | 9.9±3.6 | 10,9±2.9 | 10,0±2.3 | 9,4±4.0 | 10,1±3.2 |
| HCT (%) | 24.2±9.2 | 26,6±7.3 | 24,2±6.1 | 24,3±10.4 | 24,8±8.5 |
| MCV (fL) | 36.9±0.4 | 37,7±0.5 | 37,4±0.2 | 37,6±0.2 | 37,4±0.5 |
| MCH (pg) | 15.2±0.5 | 15,5±0.6 | 15,6±0.8 | 14,6±0.3 | 15,2±0.1 |
| MCHC (g/dL) | 41.3±1.2 | 41,0±1.7 | 41,7±2.1 | 38,7±1 | 40,7±0.5 |
| WBC (x10^3^ µL) | 38.6±2.5 | 50,5±10 | 44,9±37.6 | 44,4±14.9 | 49,5±3.4 |
| Platelets(x10^3^ µL) | 237,8±203 | 272,7±216 | 234,8±179 | 281,6±218 | 231,7±284 |
| ALT (U/I) | 24±0.8 | 26.6±1.2 | 21±7 | 29.9±0.1 | 26.5±2.9 |
| Creatinine (mg/dL) | 0.3±0.3 | 0.6±0.2 | 0.4±0.1 | 0.5±0.2 | 0.2±0.2 |
| Urea (mg/dL) | 41±3.2 | 48.7±5.2 | 43±0.2 | 42.7±5.2 | 49.3±2.4 |

**Legend:** WBC White Blood Cells; RBC Red Blood Cells; HGB Hemoglobin; HCT Hematocrit; MCV: Mean corpuscular volume, MCH: Mean corpuscular hemoglobin, MCHC: Mean corpuscular hemoglobin concentration, WBC: White Blood cells, ALT alanine aminotransferase.  Values are represented as mean ± standard error.
